# Central nervous system manifestations following vaccination against COVID-19

**DOI:** 10.1016/j.bbih.2024.100788

**Published:** 2024-05-03

**Authors:** Seyed Sepehr Khatami, Mona-Elisabeth Revheim, Poul Flemming Høilund-Carlsen, Abass Alavi, Samaneh Ghorbani Shirkouhi, Sasan Andalib

**Affiliations:** a Department of Neurology, University of California Irvine, California, USA; b The Intervention Center, Division of Technology and Innovation, Oslo University Hospital, Oslo, Norway; c Institute of Clinical Medicine, University of Oslo, Oslo, Norway; d Department of Nuclear Medicine, Odense University Hospital, University of Southern Denmark, Odense, Denmark; e Research Unit of Clinical Physiology and Nuclear Medicine, Department of Clinical Research, Faculty of Health Sciences, University of Southern Denmark, Odense, Denmark; f Department of Radiology, Perelman School of Medicine, University of Pennsylvania, Pennsylvania, USA; g School of Medicine, Shahroud University of Medical Sciences, Shahroud, Iran; h Research Unit of Neurology, Department of Clinical Research, Faculty of Health Sciences, University of Southern Denmark, Odense, Denmark; i Department of Neurology, Odense University Hospital, Odense, Denmark

**Keywords:** Central nervous system, Neurology, Complications, COVID-19, SARS-CoV-2, Vaccination

## Abstract

Coronavirus disease 2019 (COVID-19) vaccination has become the most effective countermeasure in the severe acute respiratory syndrome coronavirus 2 (SARS-CoV-2) pandemic. However, vaccination is associated with side effects. This narrative review focuses on central nervous system (CNS) manifestations following COVID-19 vaccination and provides a summary of the potential underlying mechanisms and methods of diagnosis and management of the vaccination-related CNS manifestations. Headache, myalgia, optic neuritis, seizure, multiple sclerosis, acute disseminated encephalomyelitis and encephalitis, delirium, acute transverse myelitis, and stroke have been reported after COVID-19 vaccination. Constant headache and myalgia are common manifestations that may necessitate further clinical investigation for stroke. To limit consequences, it is imperative to follow standard treatment protocols for each neurological disorder following COVID-19 vaccination. Immunosuppressive medication can be helpful in the treatment of seizures following vaccination since the immune response is involved in their etiology. Clinicians should be aware of the manifestations after COVID-19 vaccination to respond promptly and effectively. Clinical guidelines for the management of CNS manifestations following COVID-19 vaccination are in high demand and would be useful in each new SARS-CoV-2 variant pandemic.

## Highlights

• Vaccination has been shown to be effective against COVID-19.
• Central nervous system complications may arise from COVID-19 vaccination.
• Knowing these manifestations is a crucial prerequisite for prompt and effective therapy.

## Abbreviations

ACE2: Angiotensin-Converting Enzyme 2
ADEM: Acute Disseminated Encephalomyelitis
ATM: Acute Transverse Myelitis
COVID-19: Coronavirus Disease 2019
CSF: Cerebrospinal Fluid
CT: Computed tomography
CVST: Cerebral Venous Sinus Thrombosis
CNS: Central Nervous System
EEG: Electroencephalogram
FDG: Fluorodeoxyglucose
IVIG: Intravenous Immunoglobulin
MOG: Myelin Oligodendrocyte Glycoprotein
MRI: Magnetic Resonance Imaging
mRNA: Messenger Ribonucleic Acid
MRNA-1273: Moderna Vaccine
MS: Multiple Sclerosis
NSAIDs: Non-steroidal Anti-inflammatory Drugs
PET: Positron Emission Tomography
PF4: Platelet Factor 4
SARS-CoV- 2: Severe Acute Respiratory Syndrome Coronavirus 2
VAERS: Vaccine Adverse Event Reporting System
VITT: Vaccine-Induced Immune Thrombotic Thrombocytopenia

## 1 Introduction

COVID-19, which stands for Coronavirus Disease 2019, is caused by Severe Acute Respiratory Syndrome Coronavirus 2 (SARS-CoV-2). So far, COVID-19 has contaminated over 769 million individuals worldwide, with approximately 7 million mortalities (Najafi et al., 2022; World-Health-Organization, 2023). The spike (S) protein of the virus has a high affinity to the angiotensin-converting enzyme 2 (ACE2) receptor, which is essential for its endocytosis and quick spread in individuals. Effective vaccination remains the most reliable method to prevent the disease (Umakanthan et al., 2021). The approved vaccines against COVID-19 are efficient in preventing both severe and moderate types of the disease (Bloem et al., 2021). Inactivated or live-attenuated viruses, recombinant proteins, and vector technologies are used to develop COVID-19 vaccines (Aladdin and Shirah, 2021). Johnson & Johnson's COVID-19 Vaccine (Ad26.COV2.S) generates the full-length spike glycoprotein by employing a replication-defective adenovirus vector. Oxford-AstraZeneca (ChAdOx1 nCoV-19 or AZD1222) and Sputnik V (rAd26-S and rAd5-S) are also vector-based COVID-19 vaccines. Additionally, there are two messenger ribonucleic acid (mRNA) vaccines. Moderna vaccine (mRNA-1273) encodes a full-length S protein, and Pfizer-BioNTech (BNT162b2) vaccine expresses a receptor-binding domain (Jackson et al., 2020; Lotan et al., 2021). Sinopharm is an inactivated SARS-CoV-2 virus vaccine (Chen et al., 2022). Severe health consequences associated with COVID-19 vaccines are uncommon, while some of the consequences may occur in vaccinated individuals by chance (Gubernot et al., 2021). However, concerns about cerebral venous sinus thrombosis (CVST) and thrombocytopenia following COVID-19 vaccinations halted the distribution of Johnson & Johnson's vaccine in the United States (Shay et al., 2021). Increased rates of venous thromboembolic events, including CVST, were observed in individuals receiving Oxford-AstraZeneca in Denmark and Norway (Pottegård et al., 2021).

There is a growing body of literature indicating neurological manifestations following COVID-19 vaccination. These manifestations mostly occur in women (∼64%) with a median age of 50 years (Alonso Castillo and Martínez Castrillo, 2022). A better understanding of these side effects of COVID-19 vaccination is essential in the clinical setting to allow prompt and effective management.

**Fig. 1:**
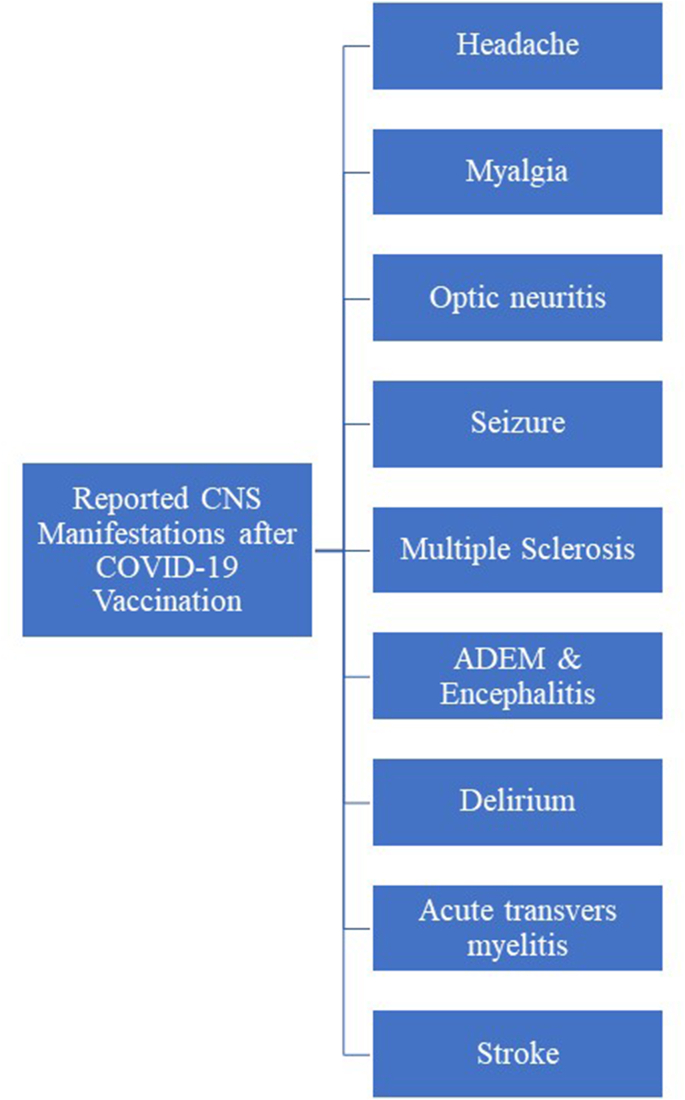
Overview of reported CNS manifestations after COVID-19 vaccination. Note: CNS: Central Nervous System; COVID-19: Coronavirus Disease 2019; ADEM: Acute Disseminated Encephalomyelitis.

In this narrative review, we present the central nervous system (CNS) manifestations following vaccination against COVID-19, as well as an overview of underlying mechanisms, diagnosis, and management of each disease/condition after the vaccinations. Fig. 1 shows an overview of the reported CNS manifestations after COVID-19 vaccination.

### 1.1 Headache

Headache is one of the most common adverse effects of COVID-19 vaccination. Several studies have reported headaches after the first and second vaccine doses (Costa Clemens et al., 2022; Göbel et al., 2021; Hause et al., 2022; Polack et al., 2020; Ramasamy et al., 2021; Song et al., 2021). The observed frequencies of headaches after the first dose were 52%, 55.1%, and 72% in studies by Polack et al. (2020), Göbel et al. (2021), and Song et al. (2021), respectively. The time of onset of the reported symptoms varied from 18 to 27 h after vaccination with a duration of 14.2±21.4 h (Göbel et al., 2021). A meta-analysis showed that headache has migraine-like features with pulsating quality, phono- and photo-phobia in approximately one-third of the patients receiving COVID-19 vaccines (Castaldo et al., 2022). Even though headaches in most cases are often self-limited, they can be an initial symptom of severe vaccine-related complications such as subarachnoid hemorrhage (Abdollahi et al., 2022), intracerebral hemorrhage (García-Azorín et al., 2021), and CVST (García-Azorín et al., 2021). Thus, patients should undergo computed tomography (CT) or magnetic resonance imaging (MRI) to rule out serious diseases if they have long-lasting headaches, i.e., headaches that persist more than a week after the vaccination (García-Azorín et al., 2021). Patients should use acetaminophen with or without non-steroidal anti-inflammatory drugs (NSAIDs) to manage COVID-19 vaccination side effects including headaches, according to the Centers for Disease Control and Prevention (Gelfand and Poland, 2021).

### 1.2 Myalgia

Another common CNS complication after COVID-19 vaccination is myalgia (Hyun et al., 2021; Polack et al., 2020; Ramasamy et al., 2021). A meta-analysis showed that myalgia together with headache were the most common symptoms after the COVID-19 vaccination (Chen et al., 2021). According to the meta-analysis, myalgia and headaches were more common in younger individuals than in older adults. The mechanism of myalgia after COVID-19 vaccination is not clear; yet it may arise from an immediate inflammatory production of cytokines such as IL-6, IL-12, tumor necrosis factor, and tissue damage (Giannotta et al., 2023). Surprisingly, Hyun et al. (2021) reported five cases (mean age 67 years) of polyarthralgia and myalgia syndrome after Oxford-AstraZeneca vaccination and symptoms persisting for more than 47 days, despite prednisolone and antipyretic treatment. Mostly, myalgia can be resolved without any intervention, and treatment with paracetamol (acetaminophen) does not make any difference (Chen et al., 2021). However, myalgia should be considered an initial symptom of severe disease, if it lasts longer than a few days or a week (Cantarelli Rodrigues et al., 2021).

### 1.3 Optic neuritis

The pathophysiology of optic neuritis following vaccination is not well understood yet; however, it is hypothesized that vaccination may induce an immune-mediated response against optic nerve antigens (Kerrison et al., 2002). A previous study showed that the likelihood of optic neuritis increased following COVID-19 vaccination compared to the pre-vaccine period (OR: 2.09, 95% CI = 1.93–2.27) (Zhao et al., 2024). A recent study indicates that the incidence rate of optic neuritis post-COVID-19 vaccination in the UK stands at 3.74 per 100,000 individuals (Alvarez et al., 2021). This study also shows that the rate surpasses the worldwide incidence rate of optic neuritis following vaccination, which is reported to be 0.0017 per 100,000 individuals (Alvarez et al., 2021). In a systematic review study, 26 patients (aged 19–65 years) with optic neuritis following COVID-19 vaccination were reviewed (Elnahry et al., 2022). According to this study, the onset of symptoms occurred 0–42 days after vaccination. Most of the case studies showed bilateral or left-sided optic neuritis (Arnao et al., 2022; Badrawi et al., 2021; García-Estrada et al., 2022; Helmchen et al., 2022; Jarius et al., 2022; Leber et al., 2021; Liu and Lee, 2022; Roy et al., 2022). However, in the studies of Wang et al. (2022), Donaldson and Margolin (2023), and Tugizova et al. (2023) also reported right-side optic neuritis after COVID-19 vaccination. In most of the studies of optic neuritis following COVID-19 immunization, corticosteroids were prescribed for the patients (Arnao et al., 2022; García-Estrada et al., 2022; Helmchen et al., 2022; Jarius et al., 2022; Leber et al., 2021; Liu and Lee, 2022; Roy et al., 2022), except for conservative therapy for a patient with myelin oligodendrocyte glycoprotein (MOG) (Donaldson and Margolin, 2023), intravenous immunoglobulin (IVIG) for a patient with right-side optic neuritis (Tugizova et al., 2023), and plasmapheresis for a patient with neuromyelitis optica spectrum disorder (Badrawi et al., 2021).

The first step in diagnosing optic neuritis is to obtain a contrast-enhanced MRI of the brain and orbits (Optic-Neuritis-Study-Group, 2008), as well as cerebrospinal fluid (CSF) analysis and laboratory tests to rule out inflammatory conditions or secondary infectious (Frederiksen, 1998). The recommended treatment for acute optic neuritis is intravenous methylprednisolone 1 g/day for 3–5 days, especially in cases with chronic relapsing inflammatory optic neuropathy or MOG, where it may cause dramatic visual improvement (Phuljhele et al., 2021). When there is suspicion of neuromyelitis optica spectrum disorders with aquaporin-4 IgG positivity and an inadequate response to intravenous steroids, plasma exchange or IVIG should be administered immediately (Phuljhele et al., 2021).

### 1.4 Seizure

Vaccination was discovered to be one of the causes of febrile seizures (Duffy et al., 2016). However, there is no study reporting febrile seizures as a side effect of COVID-19 vaccination, probably due to the limited use of COVID-19 vaccination in children. A cross-sectional study by Özdemir et al. (2022) reported no significant difference between the number of seizures before and after the COVID-19 vaccination, or between the two doses. According to a self-controlled case-series study (Wan et al., 2022), seizures following Pfizer-BioNTech and CoronaVac vaccinations were 1.04 and 1.11 per 100000 doses, respectively. It was noted that there was no significant difference between the two types of vaccines in terms of seizure incidence. Events reported to the Vaccine Adverse Event Reporting System (VAERS) included 8 children aged 6 months to 5 years with seizure following BNT162b2 or mRNA-1273 COVID-19 vaccination in the USA between June 18 and August 21 (Hause et al., 2022). In a study by Makhlouf et al. (2021), a case of status epilepticus after COVID-19 vaccination was reported in a patient with a medical history of treatment-resistant schizophrenia, and his symptoms finally resolved with Midazolam. Aladdin and Shirah (2021) reported a case of status epilepticus after COVID-19 vaccination in a patient who was intubated and placed in a deep coma; her symptoms markedly improved after five days of corticosteroid pulse and two sessions of plasma exchange. Electroencephalograms were generally unremarkable in some reports of seizures following COVID-19 vaccination (Ghosh et al., 2021; Ozgen Kenangil et al., 2021; Phua et al., 2021), except for a few non-significant diffuse slowing waves reported in the studies of Aladdin and Shirah (2021) and Makhlouf et al. (2021). Among the patients with seizures after COVID-19 vaccination, hippocampal atrophy was noted bilaterally in two studies (Aladdin and Shirah, 2021; Phua et al., 2021), and left-sided in one study (Makhlouf et al., 2021). Moreover, periventricular leukoaraiosis and diffuse cortical atrophy were reported in the study of Ghosh et al. (2021).

Although there is no clear explanation for the mechanism of seizures following COVID-19 vaccination, thrombotic events and excessive platelet aggregation can precipitate CVST leading to seizures (Greinacher et al., 2021; Lindgren et al., 2020). Another possible explanation for neuronal hyperexcitation and subsequent seizures is the inflammatory process (Aladdin and Shirah, 2021). Besides, the responsiveness of a patient to anti-inflammatory medications instead of antiepileptic treatments showed the importance of the inflammatory mechanism of seizure. A study by Shah et al. (2022) indicated a higher chance of seizures after COVID-19 vaccination in patients with a brain tumor. Patients with epilepsy are concerned about the COVID-19 vaccination due to fear of aggravated epilepsy symptoms (Li et al., 2021; Qiao et al., 2021). Fang et al. (2021) highlighted the increased risk of seizure following COVID-19 vaccination in patients with epilepsy, who lowered or ceased their anti-seizure medications. In contrast, von Wrede et al. (2021) pointed to the safety of COVID-19 vaccination in people with epilepsy. Steroids and plasmapheresis are recommended to be used in combination with antiepileptic medications for refractory seizures following COVID-19 immunization (Aladdin and Shirah, 2021).

### 1.5 Multiple sclerosis

Two challenges of Multiple Sclerosis (MS) patients receiving COVID-19 vaccination are relapse of existing MS (Khayat-Khoei et al., 2022) and lack of immune response due to anti-CD20 treatment (Reder et al., 2021). A previous systematic review revealed that MS following vaccination was mostly reported in women aged 37.8 ± 10.1 years (Mirmosayyeb et al., 2023). According to this study, the average time for symptoms to begin after the first and the second dose was 7.5 ± 4.8 days and 15.1 ± 12.8 days, respectively. A few studies have reported new-onset MS following vaccination against COVID-19 (Khayat-Khoei et al., 2022; Maniscalco et al., 2021; Nistri et al., 2021; Toljan et al., 2022). A retrospective study of 250 patients with MS showed that the rate of pseudo-relapses after the first, second, and third doses of BNT162b2 were 1%, 4%, and 3%, respectively (Labani et al., 2023). According to this study, the pseudo-relapse rate were 2%, 5%, and 3% following the first, second, and third doses of the mRNA-1273 vaccine, respectively; and no MS relapses were seen after the Ad26.COV2.S vaccine. Moreover, visual problems (Fragoso et al., 2022; Khayat-Khoei et al., 2022; Nistri et al., 2021), ataxia (Etemadifar et al., 2021; Fragoso et al., 2022; Khayat-Khoei et al., 2022; Nistri et al., 2021), hemiplegia (Etemadifar et al., 2021; Fragoso et al., 2022; Nistri et al., 2021; Seyed Ahadi et al., 2021), and sensory impairment (Fragoso et al., 2022; Havla et al., 2022; Khayat-Khoei et al., 2022; Łagosz et al., 2022; Maniscalco et al., 2021; Nistri et al., 2021) were the most commonly reported symptoms in the studies of MS after COVID-19 vaccination. Oligoclonal bands in the CSF of MS cases after COVID-19 vaccination were noted in a few studies (Havla et al., 2022; Khayat-Khoei et al., 2022; Nistri et al., 2021; Toljan et al., 2022). Corticosteroids were the main treatment for MS following the COVID-19 vaccination (Etemadifar et al., 2021; Fragoso et al., 2022; Havla et al., 2022; Khayat-Khoei et al., 2022; Łagosz et al., 2022; Maniscalco et al., 2021; Nistri et al., 2021; Seyed Ahadi et al., 2021), but in the studies of Toljan et al. (2022) and Havla et al. (2022), a few patients received plasmapheresis treatment as well.

Achiron et al. (2021) showed that there is no associated risk of MS relapse after the first and second doses of the Pfizer-BioNTech COVID-19 vaccine. In addition, there was no increase in the risk of acute MS relapse after the third dose of Pfizer-BioNTech vaccination, compared to the second dose (Dreyer-Alster et al., 2022). A previous study highlighted that the optimal approach to enhance antibody responses in patients with MS is to have at least a four-to-six-month gap between monoclonal antibody therapy and the first dose of the Pfizer-BioNTech vaccine (Mado et al., 2021). A gap of four to six weeks for the second dose should be considered as well (Mado et al., 2021).

### 1.6 Acute disseminated encephalomyelitis (ADEM) and encephalitis

A previous study showed that the estimated incidence rate of encephalitis post-vaccination is approximately 8 per 10 million vaccine doses for ChAdOx1 nCoV-19 based on data from multiple public databases (Zuhorn et al., 2021). Moreover, for the BNT162b2 vaccine, the incidence rate was estimated to be roughly 0.02 per 100,000 doses (Zuhorn et al., 2021). Nabizadeh et al. (2023) reviewed acute disseminated encephalomyelitis (ADEM) cases after COVID-19 vaccination in a systematic review involving mostly women aged 19–88 years. Oxford-AstraZeneca vaccine was administered to the majority of the patients in this study, and symptoms were observed from 12 h to 63 days after immunization. Gao et al. (2022) reviewed four studies including four patients with encephalitis and two patients with ADEM following COVID-19 vaccination. Moreover, Oxford-AstraZeneca vaccine was administered in half of the cases, and pleocytosis in CSF was observed in all of them. However, seizures were not reported.

Kobayashi et al. (2022) and Kwon and Kim (2022) reported encephalitis after the second dose, and Sluyts et al. (2022) reported encephalitis after the third vaccine dose. Fever (Ohara et al., 2022; Torrealba-Acosta et al., 2021), headache (Ohara et al., 2022; Torrealba-Acosta et al., 2021), decreased level of consciousness (Li et al., 2022), memory disturbance (Ohara et al., 2022), visional disturbance (Kobayashi et al., 2022; Ohara et al., 2022; Torrealba-Acosta et al., 2021), seizure (Gao et al., 2022), dysarthria (Ohara et al., 2022), and extrapyramidal symptoms (Gao et al., 2022) were the most commonly observed symptoms in the reports of encephalitis following COVID-19 vaccination. Most of the reports of encephalitis after COVID-19 vaccination show improvement after treatment with corticosteroids (Gao et al., 2022; Kobayashi et al., 2022; Li et al., 2022; Ohara et al., 2022; Torrealba-Acosta et al., 2021), while a few studies noted remaining symptoms even after that treatment (Kwon and Kim, 2022; Shin et al., 2022; Sluyts et al., 2022). Shin et al. (2022) reported empiric treatment with intravenous methylprednisolone in a patient with encephalitis after COVID-19 immunization. The most significant step in managing encephalitis is to detect the disease at an early stage through physical examination and diagnostic tests, such as MRI, CT, electroencephalography (EEG), and lumbar puncture (Abboud et al., 2021).

### 1.7 Delirium

Significant disorientation may occur following vaccine injection due to the influence of systemic inflammation on the brain, which is generally known as aseptic encephalopathy (Boutros and Keck, 1993). A study demonstrated that there was no increase in the incidence rate of delirium following vaccination with CoronaVac and BNT162b2, compared to the period before the COVID-19 pandemic (Cheung et al., 2023). This study included patients with a past history of dementia. Of the individuals in the study, 14,719 received a first dose of CoronaVac and 2730 received a first dose of BNT162b2. Subsequently, 8567 of the CoronaVac recipients and 2138 of the BNT162b2 recipients proceeded to receive a second dose. Additionally, 872 individuals received a third dose of CoronaVac, while 498 received a third dose of BNT162b2. Delirium following the vaccination were reported in six (0.04%) cases after the first dose of CoronaVac, four (0.05%) cases after the second dose of CoronaVac, and one (0.20%) case after the third dose of BNT162b2 (Cheung et al., 2023). In a case series study, 10% of older adults in nursing homes with a mean age of 82 ± 7 years developed delirium the day after receiving COVID-19 vaccination (Mak et al., 2022). More to the point, a total of 39 patients received their third shot, and physical and cognitive impairment were the most prevalent underlying conditions. There have been some reports of delirium following the Pfizer-BioNTech (Erro et al., 2021; Zavala-Jonguitud and Pérez-García, 2021), CoronaVac (Naharci and Tasci, 2021), and Ad26.COV2.S vaccines (Rivera et al., 2022). In general, the majority of therapies for delirium are symptomatic, including the use of antipsychotics and the control of fluid and electrolyte imbalances (Markowitz and Narasimhan, 2008).

### 1.8 Acute transverse myelitis (ATM)

Acute transverse myelitis (ATM) is a rare consequence of vaccines, in which polyclonal activation of B lymphocytes is a mechanism proposed for the development of ATM following vaccination (Agmon-Levin et al., 2009). A total of 119 cases of ATM were reported as post-vaccination complications based on data from the United States VAERS from 1985 to 2017 (Shah et al., 2018). Conversely, out of 51,755,447 doses of COVID-19 vaccines administered up until March 2021, with 9442 reported cases of adverse events, only 9 cases of ATM were reported (Goss et al., 2021). A systematic review of 31 patients with ATM following COVID-19 vaccination was conducted in a previous study, with the majority of patients vaccinated with Oxford-AstraZeneca, mostly women aged 52 ± 19 years (Ostovan et al., 2022). Lower extremity weakness (Alabkal et al., 2021; Alshararni, 2021; Cabral et al., 2022; Corrêa et al., 2021; Eom et al., 2022; Erdem et al., 2021; Esechie et al., 2023; Fujikawa et al., 2021; Hirose et al., 2021; Hsiao et al., 2021; Khan et al., 2022a; Mărginean et al., 2022; Maroufi et al., 2022; McLean and Trefts, 2021; Miyaue et al., 2022; Nakano et al., 2022; Sepahvand et al., 2022; Sriwastava et al., 2021; Tahir et al., 2021; Tan et al., 2021), hypoesthesia (Alabkal et al., 2021; Eom et al., 2022; Erdem et al., 2021; Esechie et al., 2023; Fitzsimmons and Nance, 2021; Fujikawa et al., 2021; Gao et al., 2021; Hirose et al., 2021; Hsiao et al., 2021; Malhotra et al., 2021; Maroufi et al., 2022; McLean and Trefts, 2021; Miyaue et al., 2022; Nakano et al., 2022; Notghi et al., 2021; Sepahvand et al., 2022; Tahir et al., 2021; Tan et al., 2021; Vegezzi et al., 2021), and abnormal deep tendon reflexes (Alabkal et al., 2021; Cabral et al., 2022; Eom et al., 2022; Hsiao et al., 2021; Khan et al., 2022a; Malhotra et al., 2021; Mărginean et al., 2022; Maroufi et al., 2022; McLean and Trefts, 2021; Notghi et al., 2021; Sriwastava et al., 2021; Tahir et al., 2021; Tan et al., 2021) were the most commonly reported symptoms in the studies of ATM following COVID-19 vaccination. Pleocytosis and elevated protein in CSF were also found in the majority of the reported cases (Alabkal et al., 2021; Albokhari et al., 2021; Alshararni, 2021; Cabral et al., 2022; Corrêa et al., 2021; Gao et al., 2021; Hirose et al., 2021; Hsiao et al., 2021; Malhotra et al., 2021; Maroufi et al., 2022; Miyaue et al., 2022; Nakano et al., 2022; Notghi et al., 2021; Pagenkopf and Südmeyer, 2021; Sriwastava et al., 2022; Tahir et al., 2021; Tan et al., 2021; Vegezzi et al., 2021); a few reports indicated unremarkable CSF (Eom et al., 2022; Fitzsimmons and Nance, 2021; Fujikawa et al., 2021; Khan et al., 2022a, 2022b; Mărginean et al., 2022; Sepahvand et al., 2022; Sriwastava et al., 2021). In addition, hyperintense lesions were observed in cervical, thoracic, and lumbar MRI scans of patients with ATM following COVID-19 vaccination (Alabkal et al., 2021; Albokhari et al., 2021; Alshararni, 2021; Corrêa et al., 2021; Eom et al., 2022; Erdem et al., 2021; Esechie et al., 2023; Fitzsimmons and Nance, 2021; Fujikawa et al., 2021; Gao et al., 2021; Hirose et al., 2021; Hsiao et al., 2021; Khan et al., 2022a, 2022b; Malhotra et al., 2021; Mărginean et al., 2022; Maroufi et al., 2022; McLean and Trefts, 2021; Miyaue et al., 2022; Nakano et al., 2022; Notghi et al., 2021; Pagenkopf and Südmeyer, 2021; Sepahvand et al., 2022; Sriwastava et al., 2022; Tahir et al., 2021; Tan et al., 2021; Vegezzi et al., 2021), except in a report of Cabral et al. (2022), in which the MRI was normal. Intravenous methylprednisolone was the main treatment in the majority of ATM case reports after COVID-19 vaccination, while plasmapheresis and IVIG were alternative treatments (Alabkal et al., 2021; Albokhari et al., 2021; Alshararni, 2021; Corrêa et al., 2021; Eom et al., 2022; Fitzsimmons and Nance, 2021; Fujikawa et al., 2021; Gao et al., 2021; Hirose et al., 2021; Hsiao et al., 2021; Khan et al., 2022a, 2022b; Malhotra et al., 2021; Mărginean et al., 2022; Maroufi et al., 2022; McLean and Trefts, 2021; Miyaue et al., 2022; Nakano et al., 2022; Notghi et al., 2021; Pagenkopf and Südmeyer, 2021; Sepahvand et al., 2022; Sriwastava et al., 2022; Tahir et al., 2021; Tan et al., 2021; Vegezzi et al., 2021). Most reports of ATM after COVID-19 vaccination noted complete or partial recovery after appropriate treatment (Alabkal et al., 2021; Albokhari et al., 2021; Cabral et al., 2022; Corrêa et al., 2021; Eom et al., 2022; Fitzsimmons and Nance, 2021; Fujikawa et al., 2021; Gao et al., 2021; Hirose et al., 2021; Hsiao et al., 2021; Khan et al., 2022a, 2022b; Malhotra et al., 2021; Mărginean et al., 2022; Maroufi et al., 2022; McLean and Trefts, 2021; Notghi et al., 2021; Pagenkopf and Südmeyer, 2021; Sepahvand et al., 2022; Sriwastava et al., 2022; Tahir et al., 2021; Tan et al., 2021; Vegezzi et al., 2021). However, in a case report by Miyaue et al. (2022), the patient experienced no improvement after 70 days of hospitalization and was transferred to a rehabilitation center. In the study of Alshararni (2021), the patient was admitted to the intensive care unit. Nakano et al. (2022) reported an expired patient with the diagnosis of ATM and pneumonia following COVID-19 immunization. The patient in a study of McLean and Trefts (2021) was positive for Coxsackie B5 and Coxsackie B6. Finally, Esechie et al. (2023) reported a case of ATM following COVID-19 vaccination in a patient who was receiving an anti-PD-L1 monoclonal antibody treatment for small-cell lung cancer.

### 1.9 Stroke

Stroke can occur after COVID-19 vaccination (Kakovan et al., 2022). Ischemic (Gorenflo et al., 2023) and hemorrhagic strokes (Bjørnstad-Tuveng et al., 2021), and CVST (See et al., 2021) have been reported following COVID-19 vaccination. But the exact mechanism is not well understood.

#### 1.9.1 Ischemic and hemorrhagic strokes

A systematic review study indicated that ischemic stroke after viral vector vaccination mostly occurred due to the Oxford-AstraZeneca vaccine (30 cases), after mRNA vaccine due to Pfizer-BioNTech (7 cases), and Moderna (1 case), and whole inactivated virus vaccine due to CoronaVac (3 cases) and Sinopharm (1 case) (Kolahchi et al., 2022). According to a meta-analysis study that included 79,918,904 individuals, the incidence relative risk of ischemic stroke and hemorrhagic stroke following COVID-19 vaccination decreased to 0.82 (95% confidence interval, 0.75–0.90) and 0.75 (95% confidence interval, 0.67–0.85), respectively (Liu et al., 2023). Hemorrhagic strokes are classified as due to subarachnoid hemorrhage or intracerebral hemorrhage (Sacco et al., 2013). The majority of hemorrhagic strokes after COVID-19 vaccination occur in the setting of vaccine-induced immune thrombotic thrombocytopenia (VITT) (Schultz et al., 2021). Nevertheless, Kim and Yoo (2022) reported a rupture of an arteriovenous malformation in the right temporal lobe in a 28-year-old woman after four days of constant headache following the first dose of the Pfizer-BioNTech vaccine. A systematic review showed that intracerebral hemorrhage occurred in 35 out of 80 (43.75 %) CVST patients after COVID-19 vaccination (Jaiswal et al., 2022).

There is an association between COVID-19-related stroke and platelet factor 4 (PF4) targeted by antibodies produced after the vaccination, which is similar to heparin-induced thrombocytopenia, referred to as VITT (Scully et al., 2021). Other possible mechanisms for COVID-19-related stroke include direct binding and activation subsequently of platelets and endothelial cells by an adenoviral vector (Chander, 2021). Spike protein expression on the membrane of megakaryocytes upon entry of adenoviral vectors is another potential mechanism (Chander, 2021). Inflammatory co-signal due to the usage of ethylenediaminetetraacetic acid during vaccine manufacturing is also considered (Greinacher et al., 2021). Immune response to anti-PF4 antibodies and activation of inflammatory and endothelial cells, as well as inflammatory processes due to the attachment of soluble spike variants to ACE2 on the surface of endothelial cells in blood vessels, contribute to the risk of stroke (Greinacher et al., 2021). In addition, Kashir et al. (2022) indicated the role of neutrophil extracellular traps in facilitating thrombosis and other immune events.

#### 1.9.2 Cerebral venous sinus thrombosis (CVST)

CVST is an atypical thrombosis of the venous sinuses of the brain (Hartel et al., 2015). A systematic review indicated that the CVST occurred mainly after vaccination with Oxford-AstraZeneca (Jaiswal et al., 2022). The risk of internal jugular vein thrombosis and intracerebral hemorrhage was greater in patients with CVST after Ad26.COV.2.S than in patients receiving Oxford-AstraZeneca (Hwang et al., 2021). Overall, CVST is relatively uncommon after vaccination against COVID-19, and the vaccination does not significantly increase CVST risk (Pawlowski et al., 2021).

#### 1.9.3 Diagnosis and management of stroke following COVID-19 vaccination

Immediate care should be considered in the presence of symptoms such as visual changes, severe headache, nausea and vomiting, abdominal pain, back pain, shortness of breath, peteciae, easy bruising or bleeding, and leg pain or swelling 4–42 days following the vaccination (James et al., 2022). In the case of VITT suspicion, the D-dimer count and anti-PF4 level are the two key indicators to evaluate, in addition to routine coagulation profile testing (Elberry et al., 2022). CT scan with arterial-venous angiography or cerebral magnetic resonance angiography must be conducted if CVST following COVID-19 vaccination is suspected (Yomayusa et al., 2022). In the case of an unremarkable initial MRI in the setting of CVST after COVID-19 vaccination, a repeat MRI is recommended (Greinacher et al., 2022; Ikenberg et al., 2021).

The main difference in the treatment of VITT from other thrombotic events is the avoidance of aspirin, heparin, and transfusion of platelets is contraindicated (Greinacher et al., 2022). IVIG, corticosteroids, and non-heparin anticoagulants are recommended as the main treatments for patients with VITT (Lee et al., 2022). However, Argatroban, a direct thrombin inhibitor, is considered an optimal anticoagulant agent (Greinacher et al., 2022). IVIG and anticoagulants are safe during pregnancy and breastfeeding; nevertheless, direct oral factor Xa inhibitors are contraindicated (Greinacher et al., 2022). In the absence of IVIG, taking short-term steroids during pregnancy or lactation is safe (Greinacher et al., 2022). Non-anticoagulant therapy such as therapeutic plasma exchange (Major et al., 2022), rituximab (Henry et al., 2021), mechanical thrombectomy (Chew et al., 2022), and decompressive craniectomy (Zamboni et al.) are also suggested as possible therapeutic options for treatment of stroke after COVID-19 vaccination.

After reviewing numerous case reports of CNS manifestations, it becomes crucial to assess the overall benefit of improved population immunity. While these serious CNS side effects are concerning, it is important to recognize that they occur at a relatively low rate compared to the vast number of individuals who receive the vaccine. The advantages of widespread vaccination, including the reduction of severe COVID-19 cases, hospitalizations, and deaths, significantly outweigh the potential risks associated with these rare neurological complications. Comprehensive monitoring and prompt medical intervention can help mitigate any adverse effects, ensuring the overall well-being of the population.

## 2 Imaging techniques for evaluating CNS complications following COVID-19 vaccination

### 2.1 Strtuctural neuroimaging

CT and MRI techniques offer valuable insights into the structural changes that take place in the nervous system (Bhargava, 2019). CT utilizes X-rays to generate detailed cross-sectional images, enabling the identification of abnormalities like hemorrhages, masses, or edema (Bhargava, 2019). Due to its accessibility, cost-effectiveness, and fast results, it has become a primary diagnostic tool for patients with headache (Lee et al., 2023), optic neuritis (Matsuo and Iguchi, 2024), seizure (Fang et al., 2023), encephalitis (Sluyts et al., 2022), delirium (Rivera et al., 2022), and stroke (García-Azorín et al., 2021) following COVID-19 vaccination.

MRI employs a powerful magnetic field and radio waves to create highly detailed images of the brain and spinal cord, allowing for the detection of subtle abnormalities such as inflammation, demyelination, or vascular changes (Radue et al., 2016). It has been widely used in patients with conditions like ATM (Alabkal et al., 2021), MS (Khayat-Khoei et al., 2022), seizure (Ghosh et al., 2021), optic neuritis (Roy et al., 2022), stroke (Cascio Rizzo et al., 2022), and headache (García-Azorín et al., 2021) after COVID-19 immunization.

### 2.2 Functional neuroimaging

Despite the high spatial resolution, CT and MRI may fail to reveal abnormalities in patients experiencing neurological manifestations after COVID-19 vaccination, the reason being that structural changes take some time to develop. In such instances, functional imaging with positron emission tomography (PET) is valuable. PET/MRI with [^18^F]fluorodeoxyglucose (FDG) and [^15^O] H_2_O can assess brain metabolism, and thus neuronal function (Kennedy et al., 1975), and cerebral blood flow (Raichle et al., 1983), respectively. A previous study demonstrated significantly increased or decreased perfusion in patients with neurological symptoms after COVID-19 vaccination but with normal MRI and CT scans (Siripongsatian et al., 2022). Moreover, patients exhibiting primary symptoms like paresthesia, weakness, headache, dizziness, facial paralysis, nausea, muscle spasms, dysesthesia, and blurred vision have shown hypometabolism in the bilateral parietal cortex (Siripongsatian et al., 2022).

FDG-PET/CT imaging can play a critical role in assessing systematic manifestations of COVID-19 (Alavi et al., 2021). Considering the similarities between certain neurological symptoms observed in COVID-19 and those occurring post-COVID-19 vaccination, coupled with the demonstrated usefulness of PET imaging in detecting COVID-19 CNS damages, it is recommended to employ PET imaging techniques for the identification of potential CNS complications following COVID-19 vaccination.

## 3 Limitations

This paper is a narrative review and, thus, prone to selection bias. No systematic review and meta-analysis were carried out, and therefore the conclusion about each manifestation is an understanding from the available body of literature. Some of the papers cited in this review were case reports and case studies. While these studies are valuable for preliminary observations, they are limited in establishing causality. These studies have lower strength of evidence and a temporal association of COVID-19 vaccination and CNS events in these studies does not inherently imply a direct causal relationship, but may reflect only incidental events between vaccination and the CNS manifestations. Thus, the conclusions in the current paper should be interpreted with caution.

## 4 Conclusion

The CNS manifestations following COVID-19 vaccination include headache, myalgia, optic neuritis, seizure, MS, ADEM and encephalitis, delirium, ATM, and stroke. To ensure early detection of CNS consequences and prevent severe disease and long-term disability, healthcare providers must be well-informed about these complications. General practitioners and other health care providers who encounter patients should be aware of symptoms such as headache, facial palsy, paralysis, blurred vision, lower extremity paralysis, myalgia, and altered mental status and be ready to carry out further diagnostic work-up and quickly initiate relevant targeted treatment. If doubts remain after additional CT and/or MR imaging, functional imaging with PET/CT or PET/MRI is recommended. The aforementioned symptoms should not call into question the justification of COVID-19 vaccination, as extensive studies reported robust protection against infection with SARS-CoV-2 variants. However, this fact only increases the need to further investigate the relationship between COVID-19 vaccines and CNS side effects.

## Funding

This research did not receive any specific grant from funding agencies in the public, commercial, or not-for-profit sectors.

## CRediT authorship contribution statement

**Seyed Sepehr Khatami:** Writing – original draft, Writing – review & editing, Visualization. **Mona-Elisabeth Revheim:** Writing – review & editing. **Poul Flemming Høilund-Carlsen:** Writing – review & editing. **Abass Alavi:** Conceptualization, Writing – review & editing. **Samaneh Ghorbani Shirkouhi:** Writing – review & editing. **Sasan Andalib:** Conceptualization, Writing – original draft, Writing – review & editing.

## Declaration of competing interest

The authors declare that they have no known competing financial interests or personal relationships that could have appeared to influence the work reported in this paper.

## Data Availability

No data was used for the research described in the article.
