# Supplementary material for: Central nervous system manifestations following vaccination against COVID-19
Source: Brain Behav Immun Health. 2024 May 3;38:100788. doi: 10.1016/j.bbih.2024.100788 (PMC11137405; doi:10.1016/j.bbih.2024.100788)
Supplement: Multimedia component 1 [file mmc1.docx]

| **Author** | **Vaccine** | **N** | **Age/Sex** | **Clinical presentation** | **Imaging and lab findings** | **Treatment and outcome** |
| --- | --- | --- | --- | --- | --- | --- |
|  |  |  | **Days/Dose** |  |  |  |
| **Optic neuritis** | | | | | | |
| [Roy et al., 2022](#_ENREF_62) | OAZ | 3 | 27/F  9/First | BCVA =20/200 in LE  Left RAPD  Left optic disc swelling | AP=Enlarged blind spot  MRI=enhancement of the left optic nerve  VEP=flat wave | IVMP  Oral steroids  BCVA improved to 20/40, decrease in optic disc swelling |
|  |  |  | 48/F  5/Second | BCVA = 20/80 in LE  Left optic disc swelling  Left RAPD | OCT= Peripapillary swelling of the retina  VEP= Delayed latency and  decreased amplitude in LE  Visual field= Inferior arcuate defect | IVMP  BCVA improved to 20/30 |
|  |  |  | 40/M  12/First | BCVA = 20/200 in both eyes  Bilateral optic disc swelling | VEP= Flat waves in both eyes  AP= Bilateral generalized depression of the visual fields | Steroid therapy  BCVA improved to 20/30 in RE and 20/40 in LE |
| [García-Estrada et al., 2022](#_ENREF_22) | J&J | 1 | 19/F  7/First | Left amaurosis  Left RAPD  Left papillitis | MRI= Enhancement of the left optic nerve  CSF= + OCB | IVMP  Oral prednisolone  BCVA improved to 20/20 in LE, and papillitis was resolved |
| [Elnahry et al., 2021](#_ENREF_10) | BNT | 2 | 69/F  16/Second | Bilateral papillitis edema and peripapillary hemorrhages  Vision loss in the RE  BCVA= Count fingers in RE  Right RAPD  BCVA= 6/6 in LE | OCT= Swelling of RNFL in both eyes with intra-retinal and sub-retinal fluid in the right macula  MRI=NL  CSF= Elevated WBC and Pr  CT=NL  Visual field testing= Central and inferior arcuate defects in the RE and inferior arcuate changes in the LE | IVMP  Improvement of optic disc swelling, and vision, only  residual macular exudates |
|  | OAZ |  | 32/F  6/First | Left optic disc swelling  BCVA = 6/9 in LE  Color vision testing = 15/20 in LE  Left RAPD | MRI= Left optic nerve enhancement  OCT= Slightly increased thickness of the LE  VEP= Asymmetric delay with latency prolonged on the left side.  MRV=NL  CSF=NL | IVMP  Oral prednisone.  Improvement in optic disc swelling and vision |
| [Leber et al., 2021](#_ENREF_36) | SC | 1 | 32/F  12hr/  Second | Bilateral anterior optic neuritis concurrent with subacute thyroiditis | MOG antibody = Positive | IVMP  Oral steroids  Improvements in optic disc swelling, vision, and thyroid function within 1 week |
| [Arnao et al., 2022](#_ENREF_5) | OAZ | 1 | N/A /F | Decreased bilateral vision acuity, decreased color perceptionring bilateral scotoma | CT=NL  OCT=NL  VEP= Bilateral latency delay (greater on the left eye)  MRI= Left optic nerve enhancement | IVMP  Fully recovered |
|  |  |  | 14/First |  |  |  |
| [Badrawi et al., 2021](#_ENREF_6) | SV | 1 | 34/M | Headache and dizziness, loss of orientation, bilateral proptosis, ataxia | AQP4-IgG=Positive  CSF=Elevated WBC & Pr  MRI(Brain)=Patchy, hyperintense signal along the ependymal surface of the ventricles, thalamus, corpus callosum, and optic chiasma  MRI (Cervical) =NL | 5 sessions of plasmapheresis  Gradually improved |
|  |  |  | 21/  Second |  |  |  |
| [Helmchen et al., 2022](#_ENREF_25) | OAZ | 1 | 40/F | Binocular blindness, back pain, paraplegia, sensory deficit, incontinence,  PMH=RRMS for 21 years and Natalizumab use 8 days before vaccination. | CSF= Elevated WBC, lactate, and Pr  MRI (Brain)= Several old lesions in the corpus callosum and periventricular white matter and hyperintensity in the chiasm and optic nerves.  MRI (Spinal) = Myelitis with the maximal extent at Th7-10, medullary conus, and hyperintensity at C7-Th2  VEP= Bilateral flat wave | Steroids, Plasmapheresis Immunoadsorption  Partial recovery of vision, however sensory and motor function without improvement |
|  |  |  | 14/First |  |  |  |
| [Donaldson and Margolin, 2023](#_ENREF_9) | OAZ | 1 | 54/M | Right inferior visional defect, right optic elevation  BCVA =20/30 IN RE,  Right RAPD | Anti-MPG antibodies anti-aquaporin4 and MOG antibody =Positive | Conservative treatment  Completely recovered |
|  |  |  | 21/First |  |  |  |
| [Wang et al., 2022](#_ENREF_78) | Sinopharm | 2 | 21/F | Right RAPD  Right optic disc blurred margin | FFA =Early hyperfluorescent leakage and late enhancement of the right optic papilla  OCT= Significant thickening of the RNFL.  Automatic static perimetry =central scotoma in the right eye.  VEP= decreased amplitude in the right eye.  MRI (Brain)= Small ischemic focus in the left frontal lobe  MRI (Orbital)=Unremarkable | Peribulbar injection of tretinoin,  IVMP  Oral prednisone  Complete improvement after 1 month |
|  |  |  | 42/First  21/Second |  |  |  |
|  |  |  | 38/F | Right RAPD  Slit lamp= Right optic disc blurred borders with congestion and edema.  Color fundoscopy photographs=Retinal vein thickening | FFA =Early stage: hyperfluorescence  leakage of the right optic papilla, Late stage: enhancement  OCT = Significant thickening of  the RNFL.  VEP=Diminished amplitude, prolonged P100 wave latency.  Automatic static perimetry = centripetal narrowing of the visual field  CT (Orbital)= hypointense thickening of the right optic nerve  Anti-MOG=Positive | IVMP  Oral prednisone  Improvement after 1 month |
|  |  |  | 21/First |  |  |  |
| [Jarius et al., 2022](#_ENREF_28) | BNT | 1 | 67/M | Left eye pain, decrease visual acuity, and color desaturation,  PMH= Hypertension, benign prostate hyperplasia | MRI= Left optic nerve swelling and contrast enhancement.  VEP= Prolonged absolute and relative P100 latency, diminished amplitude in the left eye  Anti-MOG=Positive  CSF=Elevated Pr, mild lymphomonocytic pleocytosis | IVMP  Oral tapering  Complete improvement |
|  |  |  | 10/Third |  |  |  |
| [Tugizova et al., 2023](#_ENREF_75) | BNT | 2 | 65/F | Right eye vision loss, optic disc swelling Right RAPD  PMH= MTC | MRI=Right optic neuritis  CSF=Unremarkable | IVMP  IVIG  Partial improvement after 5 weeks |
|  |  |  | 5/First |  |  |  |
|  | Mod |  | 67/M | Left eye vision loss, right eye decrease of visual acuity, bilateral eye chemosis  Left RAPD  PMH=Glaucoma | MRI=Left optic neuritis | IVMP  Completed improvement in the right eye with the remaining vision loss in the left eye |
|  |  |  | 1/Second |  |  |  |
| [Liu and Lee, 2022](#_ENREF_39) | OAZ | 1 | 49/F | Painful bilateral eye movement, Right BCVA=20/30  Left BCVA=20/200  Left RAPD, bilateral disc edema | MRI=Bilateral optic nerve enhancement, more pronounced in left side  Fluorescein angiography= Bilateral disc leakage in the late phase | IVMP  Oral prednisolone  Complete improvement after 6 weeks |
|  |  |  | 14/First |  |  |  |
| [Kanungo et al., 2023](#_ENREF_29) | OAZ | 1 | 35/F | Bilateral ophthalmoplegia, Bilateral Optic disc edema, Bilateral color vision defect  Bilateral BCVA= 6/36 | MRI=Unremarkable  MRA=Unremarkable | IVMP  Oral prednisolone  Complete improvement |
|  |  |  | 5/Second |  |  |  |
| [Natung et al., 2023](#_ENREF_52) | OAZ | 1 | 44/F | Left eye blurred vision  Bilateral optic disc edema | Fluorescein angiography= Left disc leakage in the late phase  MRI= Left optic disc swelling  VEP= Bilateral Prolonged P-100 latencies  OCT = RNFL decreased thickness in both eyes  HFA= Depressed peripheral fields in both eyes with enlarged blind spot in the left eye | IVMP declined by the patient  Partial improvement |
|  |  |  | 4/Second |  |  |  |
| [Tarcha et al., 2023](#_ENREF_72) | mRNACOVID-19 vaccine (unknown type) | 1 | 22/M | Headache, sudden bilateral vision loss, bilateral optic disc edema, Bilateral RAPD | MRI=Unremarkable  VEP= Bilateral demyelinating optic neuritis  CSF=Unremarkable | IVMP,  Plasmapheresis  IV cyclophosphamide  Improvement with remaining defect of visual capacity |
|  |  |  | 10/First |  |  |  |
| **Seizure** | | | | | | |
| [Ghosh et al., 2021](#_ENREF_23) | OAZ | 1 | 68/M | Loss of consciousness, strange vocalization  PMH=HTN | MRI= Mild periventricular leukoaraiosis and diffuse cortical atrophy  EEG=NL | Brivaracetam  Discharged |
|  |  |  | 4/First |  |  |  |
| [Phua et al., 2021](#_ENREF_59) | OAZ | 1 | 18/M | Focal and generalized tonic-clonic seizure | EEG=NL  CT venogram=NL  MRI= Generalized atrophy including mild bilateral hippocampal atrophy with no evidence of sclerosis | IV Phenytoin  Oral levetiracetam  Discharged |
|  |  |  | 5/First |  |  |  |
| [Aladdin and Shirah, 2021](#_ENREF_2) | OAZ | 1 | 46/F | Continues generalized tonic-clonic seizure. | EEG= Moderate slowing of the cerebral background  MRI= There was a slight increase in bilateral hippocampi and insula, that correlated with postictal alterations | Intubated and started on midazolam and propofol to induce a deep coma, five days of pulse steroid therapy and two sessions of plasma exchange  Significantly improved |
|  |  |  | 10/First |  |  |  |
| [Makhlouf et al., 2021](#_ENREF_41) | BNT | 1 | 62/M | Generalized tonic-clonic seizure lasting for 20 minutes  PMH=Treatment resistant schizophrenia, HTN, peripheral neuropathy | MRI=FLAIR hyperintensity and mildly restricted diffusion in the left hippocampus consistent with postictal sate  EEG= Nonspecific diffuse slowing | Midazolam  Prophylactic divalproex sodium  Discharged |
|  |  |  | 3/First |  |  |  |
| [Ozgen Kenangil et al., 2021](#_ENREF_56) | SC | 1 | 46/F | Generalized tonic-clonic seizure for the first time.  PMH= Hashimoto's thyroiditis | MRI (before treatment)= distributed hyperintense lesions in the bilateral corona radiata, left thalamus, right parietal cortex, and left diencephalon on T2 and FLAIR sequences.  CSF=Unremarkable | IVMP  No significant change in the MRI after treatment |
|  |  |  | 30/Second |  |  |  |
| [Mendu et al., 2023](#_ENREF_48) | BBV152 | 1 | 15/M | Generalized tonic-clonic seizure, loss of consciousness, mild fever | Lab data= Unremarkable  ELISA= + dengue NS1 enzyme  CT =Unremarkable  MRI=Unremarkable  EEG= Occasional discharges | IV levetiracetam  Discharge with oral levetiracetam and clobazam  Improvement |
|  |  |  | 6-7hours/First |  |  |  |
| [Pakfetrat et al., 2023](#_ENREF_58) | Sinopharm | 1 | 65/M | Hallucination, fever, disorientation, seizure | CSF=Unremarkable  Lab data= Hyponatremia  MRI= Several T2 and FLAIR hyperintensities are observed across both cerebral hemispheres Elevated signal intensity along with effacement of the sulci in the right temporal lobe, which does not show post-Gadolinium enhancement, suggesting a likelihood of encephalitis | Hyponatremia treatment was started without improvement of the symptoms. The patient was then intubated.  Levetiracetam  IVMP  IVIG  Improvement |
|  |  |  | 1/Second |  |  |  |
| **Multiple sclerosis** | | | | | | |
| [Khayat-Khoei et al., 2022](#_ENREF_32) | Mod | 7 | 35/F | Right arm weakness, dysmetria, ataxia  PMH= 11 years of RRMS | MRI = New T2 hyperintense lesion in the right cerebellum enhanced with gadolinium | IVMP  Completely improved |
|  |  |  | 21/Second |  |  |  |
|  | Mod |  | 26/F | Blurred vision, right eye pain | MRI = Multiple T2 hyperintense periventricular, subcortical, posterior fossa, and spinal cord | IVMP  Completely improved |
|  |  |  | 14/Second |  |  |  |
|  | BNT |  | 24/F | Vision changed and right eye pain  PMH= 9 years of RRMS | MRI =Several new enhancing brain lesions  without any optic abnormalities | IVMP  Completely improved |
|  |  |  | 1/Second |  |  |  |
|  | BNT |  | 64/M | Paresthesia, urinary retention, ataxia | MRI=T2 hyperintensity from the cervical spinal cord to the conus, patchy gadolinium enhancement, consistent with longitudinally extensive transverse myelitis | IVMP  Partially improved |
|  |  |  | 18/First |  |  |  |
|  | BNT |  | 33/M | Blurred vision in the left eye | MRI= Active demyelinating process  CSF=OCB+, Elevated IgG | IVMP  Completely improved |
|  |  |  | 1/Second |  |  |  |
|  | Mod |  | 44/F | Right-sided weakness  PMH=14 years of MS | MRI= New enhancing lesion in the brain | IVMP  Completely improved |
|  |  |  | 6/Second |  |  |  |
|  | BNT |  | 48/F | Right eye pain, Lhermitte’s phenomenon  PMH=CIS for 8 years | MRI = Three new T2 hyperintense brain white matter lesions | Oral prednisolone  Near to complete recovery |
|  |  |  | 15/First |  |  |  |
| [Etemadifar et al., 2021](#_ENREF_15) | SV | 1 | 34/F | Ataxia, right hemiplegia  PMH= 13 years of MS | MRI= Several new juxtacortical, brainstem periventricular, and cerebellar peduncle lesions | Corticosteroid therapy  Partially improved |
|  |  |  | 3/First |  |  |  |
| [Havla et al., 2022](#_ENREF_24) | BNT | 1 | 28/F | Left abdominal neuropathic pain, sensory impairment below the T6 level, with hypoesthesia of the right abdominal wall and genital regions, and left leg paresis  PMH=MS | MRI= Enhancing lesion at the T6 level,  multiple partially confluent lesions with spatial dissemination but no Gadolinium enhancement  CSF= pleocytosis, OCB+ | IVMP  Plasma exchange  Partially improved |
|  |  |  | 6/First |  |  |  |
| [Fragoso et al., 2022](#_ENREF_17) | OAZ | 9 | 22/F | Facial paralysis, hemiparesis, ataxia  PMH= RRMS for 5 years | MRI= Tumefactive lesion | IVMP  Not yet resolved |
|  |  |  | 7/ First |  |  |  |
|  |  |  | 32/F | Left eye loss of vision and papillitis  PMH= RRMS for 2 years | MRI= New lesions in the left eye | IVMP  IVIG  Partial improvement in 3 weeks |
|  |  |  | 10/ First |  |  |  |
|  |  |  | 35/M | Unable to walk, bilateral limbs weakness  PMH= SPMS for 3 years | MRI= High lesion load, new lesions | Oral prednisone  Not yet resolved |
|  |  |  | 7/First |  |  |  |
|  |  |  | 62/F | Left eye vision loss  PMH= NMOSD for 8 years | MRI=New lesions in left optic nerve | IVMP  Resolved after 3 weeks |
|  |  |  | 7/First |  |  |  |
|  |  |  | 32/F | Right leg and foot motor, and sensitive deficits  PMH= RRMS for 6 years | MRI= New lesions | IVMP  Not yet resolved |
|  |  |  | 7/First |  |  |  |
|  |  |  | 51/M | Bilateral upper extremity hypoesthesia  PMH= PPMS for 2 years | MRI=New lesions in the cervical cord | No treatment  Not yet resolved |
|  |  |  | 25/First |  |  |  |
|  |  |  | 35/M | Incoordination of right arm and hand  PMH= RRMS for 4 years | MRI=New lesions in brainstem | IVMP  Not yet resolved |
|  |  |  | 20/First |  |  |  |
|  |  |  | 42/F | Bilateral upper extremity weakness  PMH= RRMS for 3 years | MRI=New lesions in the spinal cord, T2 level | IVMP  Resolved after 3 weeks |
|  |  |  | 15/First |  |  |  |
|  |  |  | 30/F | Right hemiparesis  PMH= RRMS for 1 year | MRI=New lesions | IVMP  Resolved after 3 weeks |
|  |  |  | 25/First |  |  |  |
| [Seyed Ahadi et al., 2021](#_ENREF_65) | Sinopharm | 1 | 42/F | Paraparesis, bilateral lower extremities weakness, mild atrophic optic nerves on fundoscopy, decrease DTRs of lower extremity, upward plantar reflex, bilateral intentional tremor  PMH=RRMS for 20 years | MRI= Several periventricular, anterior temporal, cerebral, and anterior medullary white matter hyper-intensities, nodular enhancement in brainstem plaque | IVMP |
|  |  |  | 2/First |  |  |  |
| [Łagosz et al., 2022](#_ENREF_35) | COVID-19 vaccine (unknown type) | 1 | 64/M | Numbness, worsened mobility in the arms  PMH= MS | CT= Hypodense lesion in the left frontoparietal area  Lab= elevated troponin, CRP, ESR,  ECG= ST-elevation myocardial infarction  Echocardiography= Decrease EF and pericardial effusion | Glucocorticosteroids, IV eptifibatide, oral acetylsalicylic acid, ticagrelor,  Improvement after treatment |
|  |  |  | 1/N/A |  |  |  |
| [Maniscalco et al., 2021](#_ENREF_43) | BNT | 1 | 26/F | Left upper and lower limbs paresthesia and weakness, difficulty walking, left side hyperreflexia and loss of vibrations | MRI =enhancing lesions in the frontal and temporal area | IVMP  Resolved |
|  |  |  | 2/First |  |  |  |
| [Nistri et al., 2021](#_ENREF_53) | OAZ | 16 | 45/M | Bilateral lower limbs dysesthesia  PMH= MS for 9 years | MRI=Two new lesions in the temporal gyri and a new spinal cord lesion at T3 level | Steroids |
|  |  |  | 21/First |  |  |  |
|  | OAZ |  | 48/F | Right eye visual acuity deficit | MRI= An enhancing lesion in the corpus callosum, multiple white matter unenhanced lesions, and lesions in the occipital lobe | IVMP  Significant improvement in the visual deficit |
|  |  |  | 8/First |  |  |  |
|  | OAZ |  | 54/F | Hypoesthesia below the T6 level.  PMH= MS for 28 years | MRI= One enhancing lesion in the spinal cord | IVMP  Resolved |
|  |  |  | 3/First |  |  |  |
|  | OAZ |  | 66/F | Visual disturbance and postural instability on the right limbs | MRI: Multiple lesions in the left paratrigonal and periventricular white matter.  CSF= OCB+ | IVMP  Partial improvement |
|  |  |  | 7/First |  |  |  |
|  | Mod |  | 42/F | Weakness of the left upper limb  PMH= MS for 2 years | MRI = enhancing brain lesion in the right corona radiata | No steroids |
|  |  |  | 14/First |  |  |  |
|  | Mod |  | 57/M | Bilateral lower extremity motor deficit  PMH= MS for 20 years | MRI= Enhancing pontine lesion | IVMP  Partial improvement |
|  |  |  | 14/ Second |  |  |  |
|  | BNT |  | 49/F | Numbness on the left hand and left side of her head.  PMH= MS for 8 years | MRI=Enhancing periventricular lesion and a spinal cord lesion at the C3 level | IVMP  Almost resolved |
|  |  |  | 5/First |  |  |  |
|  | BNT |  | 39/M | Left lower limb paresthesia  PMH= MS for 7 years | MRI=Three new lesions, two of which  were enhancing in the left parietal lobe and the periventricular white matter | Oral steroids  Partial improvement |
|  |  |  | 10/First |  |  |  |
|  | BNT |  | 39/F | Right upper and lower extremity dysesthesia | MRI= Enhancing lesion in the mesencephalon | IVMP  Resolved |
|  |  |  | 3/ First |  |  |  |
|  | BNT |  | 60/F | Fatigue, bilateral lower extremity numbness  PMH= MS for 23 years | MRI=one enhancing brain lesion on the left periventricular white matter | No steroids |
|  |  |  | 2/First |  |  |  |
|  | BNT |  | 30/F | Language disturbance  PMH= MS for 3 years | MRI=Two enhancing brain lesions, one in the right corona radiata and one with conspicuous edema in the left centrum semiovale | Steroids |
|  |  |  | 20/Second |  |  |  |
|  | BNT |  | 58/F | Urinary incontinence, difficulties in walking, dysphagia.  PMH= MS for 21 years | MRI= New area with ring enhancement in the white matter of the left frontal lobe | IVMP  Improvement |
|  |  |  | 3/First |  |  |  |
|  | BNT |  | 34/F | Neck pain and right arm hypoesthesia  PMH= MS for 3 months | MRI = Three brain enhancing lesions (one right posterior paraventricular and two in the left periventricular white matter) and a new unenhanced lesion on spinal cord | IVMP |
|  |  |  | 4/Second |  |  |  |
|  | BNT |  | 35/F | Left side paresthesia  PMH= MS for 16 years | MRI=Three enhancing lesions in the left temporal lobe and left centrum semiovale | Steroids |
|  |  |  | 1/Second |  |  |  |
|  | BNT |  | 54/M | Right hemiparesis.  PMH= MS for 18 years | MRI=Two ring-enhancing lesions located in the left periventricular white matter | IVMP  Resolved |
|  |  |  | 7/First |  |  |  |
|  | BNT |  | 37/M | Right side weakness  PMH= MS for 2 years | MRI=Tumefactive contrast-enhancing lesion in the left frontoparietal white matter | IVMP  Partial recovery |
|  |  |  | 10/Second |  |  |  |
| [Toljan et al., 2022](#_ENREF_73) | BNT | 5 | 29/F | left leg weakness, numbness | MRI= Multiple brain lesions in periventricular and juxtacortical white matter, with one enhancing lesion in the right centrum semiovale  CSF=Elevated Pr, Pleocytosis, +OCB | IVMP, Ocrelizumab  Improvement |
|  |  |  | 1/First |  |  |  |
|  | BNT |  | 37/M | left hand paresthesia | MRI = Multiple periventricular non-enhancing hyperintensities and a C3-C4 cord hyperintense lesion | Oral prednisolone  N/A |
|  |  |  | 3/First |  |  |  |
|  | Mod |  | 41/M | Bilateral lower extremity numbness | MRI=multiple intracranial periventricular and juxtacortical hyperintensities  CSF=Elevated Pr, Pleocytosis, +OCB | IVMP, Plasma exchange  Partial improvement with residual decreased sensation |
|  |  |  | 30/Second |  |  |  |
|  | Mod |  | 46/F | Intermittent right leg numbness (after first dose)  Left foot drop, bilateral arm pain, left lateral abdominal burning sensation (after second dose) | MRI= Periventricular and juxtacortical intracranial lesions with enhancement of the periventricular lesion.  CSF=Elevated Pr, Pleocytosis, +OCB | IVMP  N/A |
|  |  |  | N/A/First  3/Second |  |  |  |
|  | BNT |  | 43/F | Right arm weakness and right periorbital and palatal numbness  PMH=Optic neuritis | MRI= Enhancing and non-enhancing temporal and callosal periventricular ovoid lesions  CSF=+OCB | IVMP  N/A |
|  |  |  | 35/Second |  |  |  |
| [Saxton et al., 2023](#_ENREF_63) | Mod | 1 | 28/F | Decrease visual acuity of right eye, Right RAPD, reduced color perception, pain with the movement of the right eye | OCT=Unremarkable  Lab data = + JC virus, + varicella-zoster IgG  MRI= New supratentorial, infratentorial, and cord lesions | Methylprednisolone  N/A |
|  |  |  | 7/Second |  |  |  |
| **Encephalitis** | | | | | | |
| [Kobayashi et al., 2022](#_ENREF_33) | BNT | 1 | 46/F | Diplopia Bilateral abduction restrictions | MRI= Lesion on the dorsal pons across the midline and no gadolinium enhancement  MRA=NL  CSF=NL | IVMP  Oral steroid  Improved |
|  |  |  | 1/Second |  |  |  |
| [Kwon and Kim, 2022](#_ENREF_34) | OAZ | 1 | 57/F | Generalized convulsive seizure, cognitive decline, dysphasia | MRI= Restricted diffusion along the left insular and mesial temporal cortices with the corresponding hyperintensity without contrast enhancement  Second MRI after 1 month= contrast enhancement along the mesial temporal cortex  MRI (after partial improvement) = Subsidence of the contrast enhancement, encephalomalacia change in the left temporal lobe  CSF (first)=NL  CSF (second)= Pleocytosis, elevated protein, positive oligoclonal immunoglobulin G (IgG) band  EEG= Intermittent generalized rhythmic delta activity | Levetiracetam  Oxcarbazepine  Intravenous acyclovir  IVMP  Rituximab  Language function partially improved after rituximab  Memory dysfunction poorly improved |
|  |  |  | 1/Second |  |  |  |
| [Ohara et al., 2022](#_ENREF_55) | Mod | 2 | 23/M | Headache, fever, memory disturbance | MRI= High signal intensity lesions at the midline of the splenium of the corpus callosum  CSF (1)=NL  CSF (2)= pleocytosis, elevated protein | IVMP  Improved |
|  |  |  | 3/First |  |  |  |
|  |  |  | 33/F | Visual disturbance, dysarthria, left forearm tremor, dysesthesia of the mouth and distal limbs, visual agnosia | MRI= High signal intensity lesions at the midline of the splenium of the corpus callosum  CSF=NL | IVMP  Improved |
|  |  |  | 10/First |  |  |  |
| [Shin et al., 2022](#_ENREF_66) | OAZ | 1 | 35/F | Dysarthria, anxiety, fever, tachycardia,  severe rigidity in all limbs, catatonia, motor aphasia, jaw-opening dystonia, hypophonia, drooling  PMH=intellectual disability | MRI= Mild swelling of the right hippocampus without abnormal, chronic encephalomalacia in both frontoparietal lobes  EEG= Diffuse beta wave activity, with intermittent generalized delta waves  CSF=NL | IVMP  IVIG  Intravenous acyclovir  Symptoms were improved, however rigidity and dysfunction in communication remained |
|  |  |  | 5/First |  |  |  |
| [Sluyts et al., 2022](#_ENREF_67) | Mod | 1 | 48/M | Behavioral changes, agitation, mutism, left-sided neglect, paresis, bradyphrenia  PMH= sex reassignment surgery from female to male | CT=NL  EEG=NL  SPECT=NL  MRI= Small left internal capsule developmental venous anomaly  CSF= pleocytosis, elevated Pr | Empirical therapy without corticosteroid  Complete improvement except for amnesia |
|  |  |  | 6/ Third |  |  |  |
| [Torrealba-Acosta et al., 2021](#_ENREF_74) | Mod | 1 | 77/M | Fever, diffuse rash, dizziness, headache, double vision, bilateral upper limb myoclonus, irregular orofacial movements | Lab data= Increase in the level of creatine kinase, CRP, and ferritin. Positive RF and ANA  CSF= pleocytosis, elevated Pr | IVMP  Complete improvement |
|  |  |  | 2/First |  |  |  |
| [Vogrig et al., 2021](#_ENREF_77) | BNT | 1 | 56/F | Neuropathic pain, tremor, nystagmus, left upper extremity weakness, left hemiataxic gait | MRI= Hyperintensity on the ipsilateral superior cerebellar peduncle and frontal white matter with a major lesion on the left side  EEG=NL  CSF= Elevated IL-8, decrease TNF-α and IL-10. | ADEM was diagnosed  Prednisone  Improvement with remaining of mild dysmetria and intention tremor of the left upper limb |
|  |  |  | 14/First |  |  |  |
| [Li et al., 2022](#_ENREF_38) | OAZ | 1 | 55/M | Weakness, lowered consciousness, drowsiness, disorientation to people and place, slow response, GCS=10 (3-15), impaired verbal expression, + Kerning's sign, +Brudzinski's sign  PMH=HTN, hyperlipidemia, sleep apnea | CSF=Pleocytosis, elevated Pr, ANA+  Serum Lab= ANA+, elevated ferritin, elevated D-dimer  MRA (Brain)= Patchy meningeal enhancement over the brain parenchyma. | Empiric IV ceftriaxone and acyclovir  Intravenous dexamethasone  Significant improvement |
|  |  |  | 6/First |  |  |  |
| [Gao et al., 2022](#_ENREF_20) | Mod | 1 | 82/F | General discomfort, palpitations, elevated blood pressure, hand tremors, confusion, left upper limb weakness, mask-like face,  gait deviation to the right  PMH= Diabetes, hypertension | MRI (Brain)= Gyral enhancement on T1-weighted imaging of the right middle and posterior temporal lobes  MRI (Cervical)=Herniated intervertebral discs at the C3-C5 level with no evidence of myelitis  EEG =Intermittent focal slow waves in the right frontocentral to parietal regions  CSF=Elevated Pr  Somatosensory evoked potentials and brainstem auditory evoked potentials=Unremarkable  VEP=An absent P100 response in the right eye due to severe diabetic retinopathy | IV valproic acid  IV dexamethasone  Significant improvement |
|  |  |  | 15/First |  |  |  |
| [Zlotnik et al., 2022](#_ENREF_81) | BNT | 1 | 48/M | Memory deficits, anterograde amnesia | MRI= Hyper-intense signal on both medial temporal lobes including the parahyppocampal gyrus  CT scan (Abdomen)= Liver cyst, adrenal adenoma  CSF=Unremarkable  Lab data= hyponatremia, anti-LGI1 AE positive | IVMP  Improvement |
|  |  |  | 17/Second |  |  |  |
| [Lewis et al., 2023](#_ENREF_37) | BNT | 1 | 56/M | Cognitive impairment, behavioral changes, palpitation | EEG= Faciobrachial dystonic seizures and left hemisphere electrographic seizures  MRI= Signal abnormality in bilateral mesial temporal lobes, left temporal pole and left insula  CSF= Elevated protein, glucose, and IgG, + LGI1 antibodies  Lab data= Hyponatremia, elevated IL-6 | IVMP  IVIG  Oral prednisolone  Improvement |
|  |  |  | 90/Second |  |  |  |
| [Mansour et al., 2023](#_ENREF_44) | BNT | 2 | 40/F | Memory disturbance, headache, severe cognitive disorders, four episodes of febrile tonic-clonic seizures | MRI = Bitemporal encephalitis  CSF=Unremarkable | Initial treatment with acyclovir with no improvement  Transferred to ICU  IVIG  Improvement |
|  |  |  | 3/First |  |  |  |
|  | Mod |  | 35/F | Confusion, febrile tonic-clonic seizure, status epilepticus, dysautonomia | MRI= Unremarkable  CSF= Unremarkable | Initial treatment with acyclovir and Cefotaxime without improvement  Admitted to ICU  IVMP  Improvement |
|  |  |  | 21/First |  |  |  |
| [Takenaka et al., 2023](#_ENREF_70) | BNT | 1 | 25/M | Fever, altered mental status  PMH= Left parotid cancer | Lab data= Elevated liver enzyme, mild increase D-dimer, +MOG antibody  CSF= +OCB, elevated pr  MRI= high intensity at the splenium corporis callosi and low intensity on apparent diffusion coefficient  EEG= Dominant rhythm of 8 Hz and intermittent semirhythmic theta wave in the bifrontal-parietal regions | IVMP  Improvement |
|  |  |  | 2/Second |  |  |  |
| **Delirium** | | | | | | |
| [Zavala-Jonguitud and Pérez-García, 2021](#_ENREF_80) | BNT | 1 | 89/M | Confusion, fluctuating attention, anxiety and  inversion of the sleep-wake cycle  PMH= DM, HTN, CKD, BPH, hearing impairment, depression | Lab data= Unremarkable | Acetaminophen,  Quetiapine  Improvement |
|  |  |  | 2/First |  |  |  |
| [Erro et al., 2021](#_ENREF_13) | BNT | 1 | 79/F | Fever, confusion, delusions, dyskinesia  PMH= Parkinson’s disease | Lab data=Elevated D-dimmer | Paracetamol  Reduction in levodopa dose  Partial improvement with remaining confusion and dyskinesia |
|  |  |  | 1/Second |  |  |  |
| [Naharci and Tasci, 2021](#_ENREF_50) | SC | 1 | 88/F | Confusion, hallucinations, agitation, sleep disturbances  PMH= Osteoporosis, previous delirium episodes, Alzheimer’s disease | Lab data= Mild normocytic anemia, leukocyturia (2+) | Haloperidol  Trazodone  Improvement |
|  |  |  | 1/First |  |  |  |
| [Rivera et al., 2022](#_ENREF_61) | J&J | 1 | 92/F | Fever, agitation, hypoxia, tachycardia  PMH= HTN, hearing impairment, AF, anemia | Lab data= leukocytosis, low Hemoglobin, elevated ESR, elevated D-dimmer,  CXR= chronic interstitial changes  CTA=Unremarkable | Metoprolol  Ferrous sulfate  Lisinopril  Acetaminophen  Improvement |
|  |  |  | 1/- |  |  |  |
| [Galan and Shaooli, 2023](#_ENREF_19) | BNT | 1 | 76/M | Disorientation, agitation, restlessness, confusion  PMH= Bladder cancer, prostate cancer, coronary artery disease | Lab data= Unremarkable  MRI=Unremarkable | Supportive care  Improvement |
|  |  |  | 4hours/Second |  |  |  |
| **Acute transverse myelitis** | | | | | | |
| [Alabkal et al., 2021](#_ENREF_1) | BNT | 1 | 26/F | Saddle anesthesia, numbness, allodynia, decreased sensation to pinprick, temperature, and light touch in S1, S4, + Romberg | MRI= A short segment T2 hyperintense and diffusely enhancing lesion at T5  CSF= Pleocytosis and elevated IgG | IVMP  Fully recovered |
|  |  |  | 3/First |  |  |  |
| [Albokhari et al., 2021](#_ENREF_3) | BNT | 1 | 16/F | Weakness of all extremities, decrease sensation to fine and pain stimuli in the lower extremity, increased tone with spasticity pattern, and hyperreflexia, +Babinski sign | MRI= Acute inflammation on the dorsal spinal cord, including contrast of the cervical, and thoracic spinal cord | Fully recovered after 5 days of hospitalization |
|  |  |  | 2/Second |  |  |  |
| [Alshararni, 2021](#_ENREF_4) | BNT | 1 | 38/M | Headache, pain in the lower extremities, numbness, lower extremities paralysis | MRI= Acute inflammation on the spine observed on the dorsal spinal cord with contrast and lumbosacral spinal cord.  CSF= Elevated pr | Admitted to ICU |
|  |  |  | 2/First |  |  |  |
| [Cabral et al., 2022](#_ENREF_7) | BNT | 1 | 33/M | Lower extremities weakness, sensation  of incomplete emptying of the bladder and nocturnal low back pain. Decreased sensation below the T12 dermatomal level | MRI (Brain cervical, thoracic, and lumbar) =Unremarkable  CSF= Pleocytosis and elevated Pr | Gradually improved after 3 months |
|  |  |  | 2/Second |  |  |  |
| [Corrêa et al., 2021](#_ENREF_8) | OAZ | 1 | 65/M | Tetraparesia | MRI= A lesion with a hyperintense signal on T2 and short tau inversion recovery with no enhancement, cervical degenerative discopathy, the spinal cord lesion extended beyond the level of the degenerative disc was later realized to the left portion of the spinal cord, and, in the axial plane CSF=Elevated Pr | IVMP  Oral tapering  Almost complete improvement |
|  |  |  | 8/First |  |  |  |
| [Eom et al., 2022](#_ENREF_11) | BNT | 2 | 81/M | Bilateral hand weakness, numbness in his fingers.  DTR increased in the upper limbs.  PMH=HTN, DM | MRI=High signal intensity from the C1 to C3 vertebrae.  MRI (Brain)= Mild brain atrophy  CSF= Unremarkable | IVMP  Oral prednisolone  The hand weakness improved, however, he continued to have limitation of his finger movements after 1 month |
|  |  |  | 3/ Second |  |  |  |
|  | BNT |  | 23/F | Tingling in her legs, lower extremities weakness, unable to walk, urinary retention,  DTR= absent in both legs | MRI=High signal intensity lesion at the conus medullaris  MEP= Absent  SEP= prolonged in the lower limbs.  CSF=Unremarkable | IVMP  Oral prednisolone  Improvement after 3 months and able to walk |
|  |  |  | 21/First |  |  |  |
| [Esechie et al., 2023](#_ENREF_14) | BNT | 1 | N/A | Lower extremity paralysis, sensory loss from the chest down, overflow incontinence  PMH=Small cell lung cancer | MRI= Enhancing lesions from C7-T7 | IVMP  Plasma exchange for 3 days.  Increased strength in lower extremities. Sensory level remained unchanged |
|  |  |  | 1/Second |  |  |  |
| [Fitzsimmons and Nance, 2021](#_ENREF_16) | Mod | 1 | 63/M | lower back and buttocks pain, lower extremities pain and paresthesias, urinary retention  Left foot drop, | MRI= Increased T2 cord signal seen in the distal spinal cord and conus.  MRI (Brain)= Few punctate T2/FLAIR signal hyperintensities in bilateral corona radiata, nonspecific  CSF = Unremarkable  EMG = One positive sharp wave in left gastrocnemius muscle | IVMP  IVIG  Oral steroid  Improvement, remaining of numbness in lower extremities |
|  |  |  | 17 hours /Second |  |  |  |
| [Fujikawa et al., 2021](#_ENREF_18) | Mod | 1 | 46/F | Upper back pain, T10 dermatome paresthesia and bilateral weakness of the upper and lower limbs, partial urinary retention, decreased sensation to light touch from her feet to the T4 vertebra, decrease lower limbs DTR  PMH= vitamin B12 deficiency | MRI (Cervical)= Intramedullary signal increase of the central gray matter at C6-T2 without enhancement  CSF= Unremarkable  U/A and U/C=contamination | IVMP  Improved bilateral lower extremity muscle weakness after one month |
|  |  |  | 2/First |  |  |  |
| [Hirose et al., 2021](#_ENREF_26) | Mod | 1 | 70/M | Bilateral lower extremities hypoesthesia and mild paraparesis, increased DTR  PMH= HTN  hyperuricemia, alcoholic liver cirrhosis | MRI(T2-weighted) = Several high-intense areas at the Th1/2 and Th5/6 vertebral levels with weak gadolinium enhancement  MRI(Brain)= Non-specific lesions  CSF=Elevated Pr, + OCB | IVMP  Oral prednisolone  Fully recovered after 57 days |
|  |  |  | 7/ First |  |  |  |
| [Hsiao et al., 2021](#_ENREF_27) | OAZ | 1 | 41/M | Left peripheral facial palsy, a tingling sensation over the T4 dermatome, paresthesia below T4, lower extremities weakness, joint position, and vibration loss in the lower extremities, increased DTR | MRI= Intramedullary-enhancing lesion at the T1 to T6  CSF= Pleocytosis, elevated Pr | IVMP  Oral prednisolone  Fully recovered |
|  |  |  | 14/First |  |  |  |
| [Gao et al., 2021](#_ENREF_21) | Mod |  | 76/F | Bilateral lower extremities paresthesia predominantly on the right side, right upper paresthesia, gait disturbance, and sacral paresthesia, decreased sensation below the right T4 dermatome, reduced proprioceptive sensation beneath the right T4 dermatome, right side decrease DTR, + Babinski sign | MRI= Intramedullary hyperintensity at C2–C5 levels on T2-weighted images, and ring enhancement at C3 level on T1-weighted images.  MRI (Brain)=Unremarkable.  CSF=Pleocytosis, elevated Pr  SEP and NCS= Bilateral peroneal neuropathy.  MEP and VEP=NL  AEP= Right sensorineural hearing | IVMP  Oral prednisolone  hydroxocobalamin  Improvement |
|  |  |  | 6/First |  |  |  |
| [Khan et al., 2022b](#_ENREF_31) | SC | 1 | 61/F | Paresthesia and bilateral upper and lower limb weakness,  PMH= Asthma, HTN | CT (chest)= Multiple asymmetrical ground-glass opacities in both lungs.  Lab=Elevated CRP, LDH, D-dimer. Negative COVID-19 antigen test  MRI= hyperintense signal in the cervical segment  CSF= Unremarkable | IVMP  Physical therapy  The patient also diagnosed with ILD Gradually improved |
|  |  |  | 2/Second |  |  |  |
| [Khan et al., 2022a](#_ENREF_30) | Mod | 1 | 67/F | Bilateral upper and lower extremity weakness, tingling in right lower limb,  PMH=CAD, CKD, neuropathy, colon rupture with colostomy | MRI= Hyperintense lesions and cord edema from C1-C3 with patchy enhancement.  MRI(Brain)= chronic microvascular changes  CSF= Unremarkable | IVMP  Plasmapheresis for 5 days  Improvement |
|  |  |  | 1/First |  |  |  |
| [Malhotra et al., 2021](#_ENREF_42) | OAZ | 1 | 36/M | Abnormal sensation in the lower limb ascending to the trunk | MRI=Ovoid T2 hyperintense lesion in the dorsal aspect of the spinal cord at C6 and C7 vertebral levels. | IVMP  Improvement |
|  |  |  | 8/First |  |  |  |
| [Mărginean et al., 2022](#_ENREF_45) | COVID-19 vaccine (unknown type) | 1 | 15/M | Bilateral deficit, and hypotonia of the lower limbs, urinary retention, unable to defecate, DTR decreased, + Babinski signs, cutaneous abdominal reflexes absent. | MRI=T2 hypersignal on vertebral segments C2-C5, Th2-Th5, and Th7-Th11,  Lab=cryoglobulins and IgG anti-MOG antibodies  CSF= Elevated IgG | IVMP  Fully recovered |
|  |  |  | 35/Unknown |  |  |  |
| [Maroufi et al., 2022](#_ENREF_46) | OAZ | 1 | 31/F | Lower limbs paraparesis and paresthesia, urinary retention, fecal incontinence  PMH=Hyperthyroidism | MRI=Signal hyperintensity and cord expansion from T10 to L1 with heterogeneous enhancement  CSF=Pleocytosis, elevated Pr | IVMP  Oral Prednisolone  Partially improvement with left lower extremity spasticity and hyperreflexia along with a + Babinski sign |
|  |  |  | 21/First |  |  |  |
| [McLean and Trefts, 2021](#_ENREF_47) | BNT | 1 | 69/F | Asymmetric bilateral lower and upper extremity weakness with more severity on the right side  PMH=Cervical cancer, hyperlipidemia, hypothyroidism, restless leg syndrome, and right leg sciatica | MRI= Extensive T2 signal abnormalities seen particularly in the anterior aspect, as well as the mid cord, extending from C3-4 down to T2-3  Brain MRI=Unremarkable  CSF= 2 OCB with 2 matching bands in the serum.  Lab= + Coxsackie B5, + Coxsackie B6 | IVMP  Physical and occupational therapy  Remaining weakness after 2 weeks. Experienced some episodes of urinary urgency and incontinence. Able to walk with a walker |
|  |  |  | 2/First |  |  |  |
| [Miyaue et al., 2022](#_ENREF_49) | BNT | 1 | 75/M | Completely paralyzed of lower limbs, lower back pain, Reduce sensation during urination,  PMH= hyperlipidemia, HTN | MRI=Longitudinally hyperintense lesion from the lower thoracic to the lumbar spine,  MRI (Brain)=NL  CSF=Elevated Pr, Pleocytosis  NCS= Compound muscle action potentials were not evoked in the tibial or peroneal nerves | IVMP  Oral prednisolone  Plasma exchange for 7 days  No improvement after 70 days of hospitalization and transferred to the rehabilitation |
|  |  |  | 3/First |  |  |  |
| [Nakano et al., 2022](#_ENREF_51) | BNT | 1 | 85/M | Vertigo, rotatory nystagmus on the right side, bilateral lower limbs hypoesthesia, bilateral upper and lower limbs hyporeflexia without an extensor plantar response, urinary retention, gait disturbance | CSF= Pleocytosis, elevated Pr  MRI= Longitudinal hyperintense lesion from the T3–5 vertebral levels on T2-weighted  imaging  MRI (Brain)= Unremarkable  Lab=Pancytopenia elevated CRP | IVMP  After 58 days since vaccination his symptoms did not improve, and the patient died |
|  |  |  | 15/Second |  |  |  |
| [Notghi et al., 2021](#_ENREF_54) | OAZ | 1 | 58/M | Lower limbs numbness allodynia up to  neck level, genital dysesthesia, urinary incontinence, hyperesthesia below  T7, hyperreflexia in all four limbs, dysesthesia in his hands and fingers  PMH=DM, pulmonary sarcoidosis | MRI= T2-weighted hyperintense signal up to C1 level  CSF= elevated Pr, Pleocytosis  Chest CT= Calcified mediastinal lymph nodes. Nodules distributed perilymphatically and within the pulmonary fissures | IVMP  Oral prednisolone  Plasma exchange for 5 days  Improvement after the second day of plasma exchange |
|  |  |  | 7/First |  |  |  |
| [Pagenkopf and Südmeyer, 2021](#_ENREF_57) | OAZ | 1 | 45/M | Headache, thoracic back pain, general  weakness. | MRI= T2 hyperintense signal of the  spinal cord from C3 to Th2 without enhancement,  CSF= Pleocytosis, elevated Pr | IVMP  Improvement with residual mild paresis of distal finger flexors and dorsiflexion of the toes, intermittent paranesthesia in feet, and a slightly impaired bowel and bladder emptying |
|  |  |  | 8/First |  |  |  |
| [Sepahvand et al., 2022](#_ENREF_64) | Sinopharm | 1 | 71/M | Left hemiparesis, paresthesia in both hands, urinary retention, hypoesthesia in the right side of the trunk and right extremities, impaired position and vibration, +Babinski sign in the left leg  PMH=DM | MRI= Longitudinally T2-hyperintense lesion without enhancement from cervicomedullary junction to C3 level  MRI (Brain)=Unremarkable  CSF=Unremarkable | IVMP  Significant improvement |
|  |  |  | 5/First |  |  |  |
| [Sriwastava et al., 2021](#_ENREF_68) | Mod | 1 | 67/F | Lower extremity weakness, DTR decrease, difficulty ambulating | MRI= Intramedullary cord signal changes extending from C1-C3 with patchy enhancement.  MRI (Brain)= Nonspecific deep white matter changes  CSF=Unremarkable | IVIG  Plasmapheresis  Partial improvement |
|  |  |  | 1/First |  |  |  |
| [Tahir et al., 2021](#_ENREF_69) | J&J | 1 | 44/F | Back pain, urinary retention, numbness, and weakness in the lower extremities | MRI= Increased signal from C2-3 segment into the upper thoracic spine  CSF=Pleocytosis | IVMP  Plasma exchange  Bell’s palsy during plasma exchange. All symptoms resolved after the treatment |
|  |  |  | 10/- |  |  |  |
| [Tan et al., 2021](#_ENREF_71) | OAZ | 1 | 25/F | Urinary retention, bilateral lower limb weakness, numbness and allodynia below the T8 spinal level, and increased DTR at the knees and ankles with upgoing plantar | MRI= Multi-segment T2-hyperintensities (T3-T5, T7-T8, and T11-L1), variable cord enhancement at T7-T8  MRI (Brain)=Unremarkable  CSF=Elevated Pr | IVMP  Improvement with remaining numbness and pain at the distal lower extremities |
|  |  |  | 5/ First |  |  |  |
| [Vegezzi et al., 2021](#_ENREF_76) | OAZ | 1 | 44/F | Bilateral plantar feet ascending paresthesia,  decreased sensation in the lower back and during micturition | MRI= Two lesions in the posterior paramedian cord at T7-T8 level and the left lateral cord at T10-T11 level  MRI (Brain)=Unremarkable  CSF=Pleocytosis and elevated Pr | IVMP  Oral prednisolone  Full recovery after one month |
|  |  |  | 4/First |  |  |  |
| [Erdem et al., 2021](#_ENREF_12) | SC | 1 | 78/F | Tetraparesis, paresthesias of bilateral upper extremities, and urinary retention | MRI= Longitudinally extensive transverse myelitis from the C1 to the T3 | IVMP  Plasmapheresis  Recovered partially |
|  |  |  | 21/First |  |  |  |
| [Madike and Lee, 2023](#_ENREF_40) | OAZ | 1 | 79/M | Lower limbs weakness, gait dyspraxia, hypoesthesia | CSF=Elevated protein, oligoclonal band  MRI= gadolinium enhancement extending from T1 to T7 | IVMP  Oral prednisolone  Plasmapheresis  The patient expired |
|  |  |  | 2/First |  |  |  |
| [Rabbani et al., 2023](#_ENREF_60) | Mod | 1 | 60/M | Bilateral lower extremity numbness and weakness, tingling, urinary and bowel incontinence, decrease bilateral lower limb DTR, reduced lower limb vibratory sensation | MRI= longitudinally extensive hyperintense T2 signal from T8 to T12 without contrast enhancement  CSF= increased number of RBC and mild elevation of pr  Lab data= Elevated ESR, CRP, ANA, + VZV IgG and West Nile IgG | IVMP  Improvement |
|  |  |  | 6/Second |  |  |  |
| [Xiao et al., 2023](#_ENREF_79) | BNT | 1 | 78/F | Lower limb weakness, hypoesthesia | MRI= T2 hyperintensity extending from T3 to T7 along with contrast enhancement at T5–T6  CSF=Unremarkable | IVMP  Improvement |
|  |  |  | 6/Booster |  |  |  |

Table S1: Summary of literatures regarding optic neuritis, seizure, multiple sclerosis, encephalitis, delirium, and acute transverse myelitis following COVID-19 vaccination

*Note: ADEM=Acute disseminated encephalomyelitis, AE=autoimmune encephalitis, AEP=Auditory Evoked Potentials, AF=Atrial fibrillation, ANA=Antinuclear antibody, AP=Automated perimetry , AQP4-IgG=Anti-aquaporin-4 IgG, BBV152=Bharat Biotech's Covaxin, BCC=basal cell cancer, BCVA=best-corrected visual acuity, BNT=Pfizer-BioNTech, BPH=prostatic hyperplasia, C=Cervical, CAD=coronary artery disease, CIS=Clinically isolated syndrome, CKD=chronic kidney disease, COVID-19=Coronavirus disease 2019, CRP=C-reactive protein, CSF=Cerebrospinal fluid, CT=Computed tomography, CTA=Computed tomography angiography, CXR=Chest X-ray, DM=Diabetes mellitus, DTR=deep tendon reflex, ECG=Electrocardiography, EF=Ejection fraction, EEG=electroencephalogram, ESR=erythrocyte sedimentation rate, F=Female, FFA=Fundus fluorescein angiography, FLAIR= Fluid-attenuated inversion recovery, GCS=Glasgow Coma Scale, HFA=Humphrey visual field analysis, HTN=hypertension, ICU= intensive care unit, IENFD=intra-epidermal nerve fiber density, IgG=Immunoglobulin G, IL=interleukin, ILD=Interestitial lung disease, IV=Intravenous, IVMP=intravenous methylprednisolone, J&J=Johnson & Johnson/Janssen, kg=kilograms, LDH=lactate dehydrogenase, LE=Left eye,* *LGI1=leucine-rich glioma inactivated 1, M=Male, mg=milligrams, MEP=motor evoked potential, Mod=Moderna, MOG=myelin oligodendrocyte glycoprotein, MRA=Magnetic resonance angiography, MRI=Magnetic resonance Imaging, MRV=Magnetic resonance venography, MS=multiple sclerosis, MTC=Medullary thyroid carcinoma, NCS=nerve conduction study, NL=Normal, NMOSD=neuromyelitis optica spectrum disorders, OAZ=Oxford Astra Zeneca, OCB=oligoclonal bands, OCT=Optical coherence tomography, PMH=past medical history, PPMS=primary progressive MS, Pr=protein, RAPD=Relative Afferent Pupillary Defect, RBC=* *red blood cell, RE=Right eye, RF= Rheumatoid factor, RNFL=Retinal nerve fiber layer, RRMS=relapsing-remittent multiple sclerosis, SC=Sinovac-CoronaVac (COVID-19), SEP=somatosensory evoked potentials, SPECT=single-photon emission computerized tomography, SPMS=secondary progressive MS, SV=Sputnik V, Th or T=thoracic, TNF-α=tumor necrosis factor alpha, U/A=urinary albumin, U/C=urinary creatinine, UTI=Urinary tract infection, VEP=Visual Evoked Potential, VZV=* *varicella-zoster virus, WBC=white blood cell.*

**References:**

Alabkal, J., Rebchuk, A.D., Lyndon, D., Randhawa, N., 2021. Incomplete subacute transverse myelitis following vaccination with Pfizer-BioNtech COVID-19 mRNA vaccine: a case report. Cureus 13(12):e20460 DOI: 10.7759/cureus.20460.

Aladdin, Y., Shirah, B., 2021. New-onset refractory status epilepticus following the ChAdOx1 nCoV-19 vaccine. J Neuroimmunol 357:577629-577629 DOI: 10.1016/j.jneuroim.2021.577629.

Albokhari, A.A., Alsawas, A., Adnan, M.H., Alasmari, A., Aljuhani, S., Almejalli, M., Kedah, H., 2021. Acute inflammatory transverse myelitis post-Pfizer-BioNtech-COVID-19 vaccine in 16-year-old. Journal of Medical Research and Innovation 2(5):1-4 DOI: 10.14293/S2199-1006.1.SOR-.PPVXII5.v1.

Alshararni, A., 2021. Acute transverse myelitis associated with COVID-19 vaccine: a case report. International Journal of Research in Pharmaceutical Sciences 12(3):2083-2087 DOI: 10.26452/ijrps.v12i3.4818.

Arnao, V., Maimone, M.B., Perini, V., Giudice, G.L., Cottone, S., 2022. Bilateral optic neuritis after COVID vaccination. Neurological Sciences 43(5):2965-2966 DOI: 10.1007/s10072-021-05832-9.

Badrawi, N., Kumar, N., Albastaki, U., 2021. Post COVID-19 vaccination neuromyelitis optica spectrum disorder: Case report & MRI findings. Radiology Case Reports 16(12):3864-3867 DOI: <https://doi.org/10.1016/j.radcr.2021.09.033>.

Cabral, G., Gonçalves, C., Serrazina, F., Sá, F., 2022. MRI Negative Myelitis Induced by Pfizer-BioNTech COVID-19 Vaccine. J Clin Neurol 18(1):120-122 DOI: 10.3988/jcn.2022.18.1.120.

Corrêa, D.G., Cañete, L.A.Q., Dos Santos, G.A.C., de Oliveira, R.V., Brandão, C.O., da Cruz Jr, L.C.H., 2021. Neurological symptoms and neuroimaging alterations related with COVID-19 vaccine: Cause or coincidence? Clin Imaging 80:348-352 DOI: 10.1016/j.clinimag.2021.08.021.

Donaldson, L.C., Margolin, E.A., 2023. Myelin Oligodendrocyte Glycoprotein Antibody–Mediated Optic Neuritis Following COVID-19 Vaccination Journal of Neuro-Ophthalmology 43(4):e123-e125 DOI: 10.1097/wno.0000000000001482.

Elnahry, A.G., Asal, Z.B., Shaikh, N., Dennett, K., Abd Elmohsen, M.N., Elnahry, G.A., Shehab, A., Vytopil, M., Ghaffari, L., Athappilly, G.K., Ramsey, D.J., 2021. Optic neuropathy after COVID-19 vaccination: a report of two cases. International Journal of Neuroscience 133(8):901-907 DOI: 10.1080/00207454.2021.2015348.

Eom, H., Kim, S.W., Kim, M., Kim, Y.E., Kim, J.H., Shin, H.Y., Lee, H.L., 2022. Case Reports of Acute Transverse Myelitis Associated With mRNA Vaccine for COVID-19. jkms 37(7):e52-50 DOI: 10.3346/jkms.2022.37.e52.

Erdem, N., Demirci, S., Özel, T., Mamadova, K., Karaali, K., Çelik, H.T., Uslu, F.I., Özkaynak, S.S., 2021. Acute transverse myelitis after inactivated COVID-19 vaccine. Ideggyogyaszati szemle 74(7-08):273-276 DOI: 10.18071/isz.74.0273.

Erro, R., Buonomo, A.R., Barone, P., Pellecchia, M.T., 2021. Severe Dyskinesia After Administration of SARS-CoV2 mRNA Vaccine in Parkinson's Disease. Mov Disord 36(10):2219-2219 DOI: 10.1002/mds.28772.

Esechie, A., Fang, X., Banerjee, P., Rai, P., Thottempudi, N., 2023. A case report of longitudinal extensive transverse myelitis: immunotherapy related adverse effect vs. COVID-19 related immunization complications. International Journal of Neuroscience 133(10):1120-1123 DOI: 10.1080/00207454.2022.2050907.

Etemadifar, M., Sigari, A.A., Sedaghat, N., Salari, M., Nouri, H., 2021. Acute relapse and poor immunization following COVID-19 vaccination in a rituximab-treated multiple sclerosis patient. Human vaccines & immunotherapeutics 17(10):3481-3483 DOI: 10.1080/21645515.2021.1928463.

Fitzsimmons, W., Nance, C.S., 2021. Sudden onset of myelitis after COVID-19 vaccination: an under-recognized severe rare adverse event. Available at SSRN 3841558 DOI: 10.2139/ssrn.3841558.

Fragoso, Y.D., Gomes, S., Goncalves, M.V.M., Mendes Junior, E., Oliveira, B.E.S., Rocha, C.F., Santos, G., Tauil, C.B., Araujo, R.V., Peron, J.P.S., 2022. New relapse of multiple sclerosis and neuromyelitis optica as a potential adverse event of AstraZeneca AZD1222 vaccination for COVID-19. Mult Scler Relat Disord 57:103321 DOI: 10.1016/j.msard.2021.103321.

Fujikawa, P., Shah, F.A., Braford, M., Patel, K., Madey, J., 2021. Neuromyelitis optica in a healthy female after severe acute respiratory syndrome coronavirus 2 mRNA-1273 vaccine. Cureus 13(9):e17961 DOI: 10.7759/cureus.17961.

Galan, L.M.S., Shaooli, R.C., 2023. Delirium as an Adverse Reaction to BNT162b2 mRNA Vaccine from Pfizer Inc., and BioNTech. APIK Journal of Internal Medicine:10-4103 DOI: 10.4103/ajim.ajim_66_21.

Gao, J.-J., Tseng, H.-P., Lin, C.-L., Hsu, R.-F., Lee, M.-H., Liu, C.-H., 2022. Acute encephalitis after COVID-19 vaccination: A case report and literature review. Human vaccines & immunotherapeutics 18(5):2082206 DOI: 10.1080/21645515.2022.2082206.

Gao, J.-J., Tseng, H.-P., Lin, C.-L., Shiu, J.-S., Lee, M.-H., Liu, C.-H., 2021. Acute Transverse Myelitis Following COVID-19 Vaccination. Vaccines 9(9):1008 DOI: 10.3390/vaccines9091008.

García-Estrada, C., Gómez-Figueroa, E., Alban, L., Arias-Cárdenas, A., 2022. Optic neuritis after COVID-19 vaccine application. Clinical and Experimental Neuroimmunology 13(2):72-74 DOI: <https://doi.org/10.1111/cen3.12682>.

Ghosh, R., Dubey, S., Roy, D., Mandal, A., Naga, D., Benito-León, J., 2021. Focal onset non-motor seizure following COVID-19 vaccination: A mere coincidence? Diabetes Metab Syndr 15(3):1023-1024 DOI: 10.1016/j.dsx.2021.05.003.

Havla, J., Schultz, Y., Zimmermann, H., Hohlfeld, R., Danek, A., Kümpfel, T., 2022. First manifestation of multiple sclerosis after immunization with the Pfizer-BioNTech COVID-19 vaccine. J Neurol 269(1):55-58 DOI: 10.1007/s00415-021-10648-w.

Helmchen, C., Buttler, G.M., Markewitz, R., Hummel, K., Wiendl, H., Boppel, T., 2022. Acute bilateral optic/chiasm neuritis with longitudinal extensive transverse myelitis in longstanding stable multiple sclerosis following vector-based vaccination against the SARS-CoV-2. J Neurol 269(1):49-54 DOI: 10.1007/s00415-021-10647-x.

Hirose, S., Hara, M., Koda, K., Natori, N., Yokota, Y., Ninomiya, S., Nakajima, H., 2021. Acute autoimmune transverse myelitis following COVID-19 vaccination: A case report. Medicine 100(51):e28423 DOI: 10.1097/md.0000000000028423.

Hsiao, Y.-T., Tsai, M.-J., Chen, Y.-H., Hsu, C.-F., 2021. Acute Transverse Myelitis after COVID-19 Vaccination. Medicina 57(10):1010 DOI: 10.3390/medicina57101010.

Jarius, S., Bieber, N., Haas, J., Wildemann, B., 2022. MOG encephalomyelitis after vaccination against severe acute respiratory syndrome coronavirus type 2 (SARS-CoV-2): case report and comprehensive review of the literature. J Neurol 269(10):5198-5212 DOI: 10.1007/s00415-022-11194-9.

Kanungo, S., Mishra, A., Ananta, S., 2023. Bilateral optic neuritis with complete ophthalmoplegia: An extremely rare complication following COVID-19 (Covishield) vaccination. Indian Journal of Ophthalmology-Case Reports 3(4):1133-1135 DOI: 10.4103/IJO.IJO_975_23.

Khan, E., Shrestha, A.K., Colantonio, M.A., Liberio, R.N., Sriwastava, S., 2022a. Acute transverse myelitis following SARS-CoV-2 vaccination: a case report and review of literature. J Neurol 269(3):1121-1132 DOI: 10.1007/s00415-021-10785-2.

Khan, Z., Khattak, A.A., Rafiq, N., Amin, A., Abdullah, M., 2022b. Interstitial Lung Disease and Transverse Myelitis: A Possible Complication of COVID-19 Vaccine. Cureus 14(2): e21875 DOI: 10.7759/cureus.21875.

Khayat-Khoei, M., Bhattacharyya, S., Katz, J., Harrison, D., Tauhid, S., Bruso, P., Houtchens, M.K., Edwards, K.R., Bakshi, R., 2022. COVID-19 mRNA vaccination leading to CNS inflammation: a case series. J Neurol 269(3):1093-1106 DOI: 10.1007/s00415-021-10780-7.

Kobayashi, Y., Karasawa, S., Ohashi, N., Yamamoto, K., 2022. A case of encephalitis following COVID-19 vaccine. Journal of Infection and Chemotherapy 28(7):975-977 DOI: <https://doi.org/10.1016/j.jiac.2022.02.009>.

Kwon, H., Kim, T., 2022. Autoimmune encephalitis following ChAdOx1-S SARS-CoV-2 vaccination. Neurological Sciences 43(3):1487-1489 DOI: 10.1007/s10072-021-05790-2.

Łagosz, P., Biegus, J., Gruszka, E., Zymliński, R., 2022. The surprising course of multiple sclerosis relapse in a patient after SARS-CoV-2 vaccination. Kardiologia Polska (Polish Heart Journal) 80(2):237-238 DOI: 10.33963/KP.a2022.0005.

Leber, H.M., Sant’Ana, L., Konichi da Silva, N.R., Raio, M.C., Mazzeo, T.J.M.M., Endo, C.M., Nascimento, H., de Souza, C.E., 2021. Acute Thyroiditis and Bilateral Optic Neuritis following SARS-CoV-2 Vaccination with CoronaVac: A Case Report. Ocular Immunology and Inflammation 29(6):1200-1206 DOI: 10.1080/09273948.2021.1961815.

Lewis, A., Wisely, H., Bayuk, T., Ma, Y., 2023. A case of anti-LGI1 encephalitis after mRNA COVID-19 vaccination. QJM: An International Journal of Medicine 116(7): 582–583 DOI: 10.1093/qjmed/hcad060.

Li, S.Y., Chen, H.H., Liu, P.Y., Shi, Z.Y., Lin, Y.H., Tsai, C.A., Lin, S.P., 2022. Case report of acute encephalitis following the AstraZeneca COVID-19 vaccine. Int J Rheum Dis 25(8):950-956 DOI: 10.1111/1756-185X.14372.

Liu, C.C., Lee, W.A., 2022. Bilateral Optic Neuritis after COVID-19 Vaccination: A Case Report. Vaccines 10(11):1889 DOI: 10.3390/vaccines10111889.

Madike, R., Lee, A., 2023. A case of acute transverse myelitis following the AstraZeneca COVID-19 vaccine. Glob J Medical Clin Case Rep 10(1):011-012 DOI: 10.17352/2455-5282.000169.

Makhlouf, A.T., Van Alphen, M.U., Manzano, G.S., Freudenreich, O., 2021. A Seizure After COVID-19 Vaccination in a Patient on Clozapine. Journal of Clinical Psychopharmacology 41(6):689-690 DOI: 10.1097/JCP.0000000000001488.

Malhotra, H.S., Gupta, P., Prabhu, V., Kumar Garg, R., Dandu, H., Agarwal, V., 2021. COVID-19 vaccination-associated myelitis. QJM: An International Journal of Medicine 114(8):591-593 DOI: 10.1093/qjmed/hcab069.

Maniscalco, G.T., Manzo, V., Di Battista, M.E., Salvatore, S., Moreggia, O., Scavone, C., Capuano, A., 2021. Severe Multiple Sclerosis Relapse After COVID-19 Vaccination: A Case Report. Front Neurol 12:721502 DOI: 10.3389/fneur.2021.721502.

Mansour, K., Chadli, Z., Rebai, J., Chaabane, A., Ben Ramdhane, H., Ben Fadhel, N., Aouam, K., Ben Fredj, N., 2023. Acute encephalitis following COVID-19 vaccines: A case series. Allergy: European Journal of Allergy and Clinical Immunology 78(Supplement 111):309-309 DOI: 10.1111/all.15616.

Mărginean, C.O., Meliț, L.E., Cucuiet, M.T., Cucuiet, M., Rațiu, M., Săsăran, M.O., 2022. COVID-19 Vaccine&mdash;A Potential Trigger for MOGAD Transverse Myelitis in a Teenager&mdash;A Case Report and a Review of the Literature. Children 9(5):674 DOI: 10.3390/children9050674.

Maroufi, S.F., Naderi Behdani, F., Rezania, F., Tanhapour Khotbehsara, S., Mirzaasgari, Z., 2022. Longitudinally extensive transverse myelitis after Covid-19 vaccination: case report and review of literature. Human vaccines & immunotherapeutics 18(1):2040239 DOI: 10.1080/21645515.2022.2040239.

McLean, P., Trefts, L., 2021. Transverse myelitis 48 hours after the administration of an mRNA COVID 19 vaccine. Neuroimmunology Reports 1:100019 DOI: <https://doi.org/10.1016/j.nerep.2021.100019>.

Mendu, S.B., Singavarapu, P.R., Kota, V., Sheri, A.R., Kotha Sr, R., SINGAVARAPU, P.R., reddy Sheri, A., 2023. Generalised Tonic-Clonic Seizure in Adolescents Following COVID-19 Vaccination: A Case Report on a Mere Co-incidence. Cureus 15(6): e40992 DOI: 10.7759/cureus.40992.

Miyaue, N., Yoshida, A., Yamanishi, Y., Tada, S., Ando, R., Hosokawa, Y., Yabe, H., Nagai, M., 2022. A case of refractory longitudinally extensive transverse myelitis after severe acute respiratory syndrome coronavirus 2 vaccination in a japanese man. Internal Medicine 61(5):8747-8721 DOI: 10.2169/internalmedicine.8747-21.

Naharci, M.I., Tasci, I., 2021. Delirium in a patient with Alzheimer's dementia following COVID-19 vaccination. Psychogeriatrics : the official journal of the Japanese Psychogeriatric Society 21(5):846-847 DOI: 10.1111/psyg.12747.

Nakano, H., Yamaguchi, K., Kawabata, K., Asakawa, M., Matsumoto, Y., 2022. Acute transverse myelitis after BNT162b2 vaccination against COVID-19: Report of a fatal case and review of the literature. J Neurol Sci 434:120102 DOI: 10.1016/j.jns.2021.120102.

Natung, T., Singh, T.A., Devi, O.S., Pandey, I., 2023. A rare case of bilateral optic neuritis post-Covishield (ChAdOx1-S [recombinant]) vaccination. Oman Journal of Ophthalmology 16(1):157–160 DOI: 10.4103/ojo.ojo_31_22.

Nistri, R., Barbuti, E., Rinaldi, V., Tufano, L., Pozzilli, V., Ianniello, A., Marinelli, F., De Luca, G., Prosperini, L., Tomassini, V., Pozzilli, C., 2021. Case Report: Multiple Sclerosis Relapses After Vaccination Against SARS-CoV2: A Series of Clinical Cases. Front Neurol 12:765954 DOI: 10.3389/fneur.2021.765954.

Notghi, A.A., Atley, J., Silva, M., 2021. Lessons of the month 1: Longitudinal extensive transverse myelitis following AstraZeneca COVID-19 vaccination. Clinical medicine (London, England) 21(5):e535-e538 DOI: 10.7861/clinmed.2021-0470.

Ohara, H., Shimizu, H., Kasamatsu, T., Kajita, A., Uno, K., Sugie, K., Kinoshita, M., 2022. Mild encephalitis/encephalopathy with reversible splenial lesion after COVID-19 vaccination (P1-1.Virtual). Neurology 98(18 Supplement):1883 DOI: 10.1212/WNL.98.18_supplement.1883.

Ozgen Kenangil, G., Ari, B.C., Guler, C., Demir, M.K., 2021. Acute disseminated encephalomyelitis-like presentation after an inactivated coronavirus vaccine. Acta Neurologica Belgica 121(4):1089-1091 DOI: 10.1007/s13760-021-01699-x.

Pagenkopf, C., Südmeyer, M., 2021. A case of longitudinally extensive transverse myelitis following vaccination against Covid-19. J Neuroimmunol 358:577606 DOI: <https://doi.org/10.1016/j.jneuroim.2021.577606>.

Pakfetrat, M., Malekmakan, L., Najafi, B., Zamani, T., Mashayekh, M., 2023. Post‐COVID‐19 vaccine acute encephalitis in an adult patient: A case report and literature review. Clinical Case Reports 11(2):e6915 DOI: 10.1002/ccr3.6915.

Phua, C.S., Bhaskar, S., Raymond, A.A., 2021. New Onset Focal Seizure Following COVID-19 Vaccination: Case Report DOI: 10.21203/rs.3.rs-975438/v1

Rabbani, B., Al-Awwad, A., Samkutty, D., Anadani, N., 2023. A Case of Transverse Myelitis After Moderna Severe Acute Respiratory Syndrome Coronavirus Vaccination. The Neurohospitalist 13(2):192-195 DOI: 10.1177/1941874422114505.

Rivera, C., Sarah Shi, D., Parinda Patel, D., Choudhry, H., 2022. John Dedousis. A Rare Case of Delirium after Johnson and Johnson’s Janssen’s Vaccination. Sch J Med Case Rep 10(3):223-225 DOI: 10.36347/sjmcr.2022.v10i03.013

Roy, M., Chandra, A., Roy, S., Shrotriya, C., 2022. Optic neuritis following COVID-19 vaccination: Coincidence or side-effect? - A case series. Indian journal of ophthalmology 70(2):679 DOI: 10.4103/ijo.IJO_2374_21.

Saxton, E., Panchasara, B., Sarangapani, S., 2023. Optic Neuritis following Second Administration of COVID-19 Vaccine: A Case Report. Case Reports in Ophthalmology 14(1):394-399 DOI: 10.1159/000531526.

Sepahvand, M., Yazdi, N., Rohani, M., Emamikhah, M., 2022. Cervical longitudinally extensive myelitis after vaccination with inactivated virus-based COVID-19 vaccine. Radiology Case Reports 17(2):303-305 DOI: <https://doi.org/10.1016/j.radcr.2021.10.053>.

Seyed Ahadi, M., Ghadiri, F., ahraian, M.A., Naser Moghadasi, A., 2021. Acute attack in a patient with multiple sclerosis 2 days after COVID vaccination: a case report. Acta Neurologica Belgica 123(1):253-254 DOI: 10.1007/s13760-021-01775-2.

Shin, H.-R., Kim, B.-K., Lee, S.-T., Kim, A., 2022. Autoimmune Encephalitis as an Adverse Event of COVID-19 Vaccination. J Clin Neurol 18(1):114-116 DOI: 10.3988/jcn.2022.18.1.114.

Sluyts, Y., Arnst, Y., Vanhemelryck, T., De Cauwer, H., 2022. COVID-19-booster vaccine-induced encephalitis. Acta Neurologica Belgica 122(2):579-581 DOI: 10.1007/s13760-022-01898-0.

Sriwastava, S., Shrestha, A.K., Khalid, S.H., Colantonio, M.A., Nwafor, D., Srivastava, S., 2021. Spectrum of Neuroimaging Findings in Post-COVID-19 Vaccination: A Case Series and Review of Literature. Neurology International 13(4):622-639 DOI: 10.3390/neurolint13040061.

Tahir, N., Koorapati, G., Prasad, S., Jeelani, H.M., Sherchan, R., Shrestha, J., Shayuk, M., 2021. SARS-CoV-2 vaccination-induced transverse myelitis. Cureus 13(7):e16624 DOI: 10.7759/cureus.16624.

Takenaka, M., Nakamori, M., Ishikawa, R., Aoki, S., Maruyama, H., 2023. Encephalopathy after COVID-19 vaccination during treatment with nivolumab: A case report. Clin Neurol Neurosurg 226:107632 DOI: 10.1016/j.clineuro.2023.107632.

Tan, W.Y., Yusof Khan, A.H.K., Mohd Yaakob, M.N., Abdul Rashid, A.M., Loh, W.C., Baharin, J., Ibrahim, A., Ismail, M.R., Inche Mat, L.N., Wan Sulaiman, W.A., Basri, H., Hoo, F.K., 2021. Longitudinal extensive transverse myelitis following ChAdOx1 nCOV-19 vaccine: a case report. BMC Neurology 21(1):395 DOI: 10.1186/s12883-021-02427-x.

Tarcha, R., Ghazal, A., Al‐Darwish, L., Abdoh, H., Kudsi, M., 2023. Optic neuritis after mRNA COVID‐19 vaccination: a case report. Clinical Case Reports 11(11):e8263 DOI: 10.1002/ccr3.8263.

Toljan, K., Amin, M., Kunchok, A., Ontaneda, D., 2022. New diagnosis of multiple sclerosis in the setting of mRNA COVID-19 vaccine exposure. J Neuroimmunol 362:577785 DOI: <https://doi.org/10.1016/j.jneuroim.2021.577785>.

Torrealba-Acosta, G., Martin, J.C., Huttenbach, Y., Garcia, C.R., Sohail, M.R., Agarwal, S.K., Wasko, C., Bershad, E.M., Hirzallah, M.I., 2021. Acute encephalitis, myoclonus and Sweet syndrome after mRNA-1273 vaccine. BMJ Case Rep 14(7):e243173 DOI: 10.1136/bcr-2021-243173.

Tugizova, M., Siegel, D.T., Huang, S., Su, E., Subramanian, P.S., Beres, S., Vora, N., 2023. Case Series: Atypical Optic Neuritis After COVID-19 Vaccination. Journal of Neuro-Ophthalmology 43(4):e120-e122 DOI: 10.1097/wno.0000000000001519.

Vegezzi, E., Ravaglia, S., Buongarzone, G., Bini, P., Diamanti, L., Gastaldi, M., Prunetti, P., Rognone, E., Marchioni, E., 2021. Acute myelitis and ChAdOx1 nCoV-19 vaccine: Casual or causal association? J Neuroimmunol 359:577686 DOI: <https://doi.org/10.1016/j.jneuroim.2021.577686>.

Vogrig, A., Janes, F., Gigli, G.L., Curcio, F., Negro, I.D., D'Agostini, S., Fabris, M., Valente, M., 2021. Acute disseminated encephalomyelitis after SARS-CoV-2 vaccination. Clin Neurol Neurosurg 208:106839-106839 DOI: 10.1016/j.clineuro.2021.106839.

Wang, J., Huang, S., Yu, Z., Zhang, S., Hou, G., Xu, S., 2022. Unilateral optic neuritis after vaccination against the coronavirus disease: two case reports. Doc Ophthalmol 145(1):65-70 DOI: 10.1007/s10633-022-09880-0.

Xiao, T.L., Zaharia, A., Al-Smadi, A.S., Murphy, C.J., 2023. Transverse myelitis following bivalent COVID-19 booster vaccine and quadrivalent seasonal influenza vaccine. Clinical and Experimental Neuroimmunology 14:138-141 DOI: 10.1111/cen3.12742.

Zavala-Jonguitud, L.F., Pérez-García, C.C., 2021. Delirium triggered by COVID-19 vaccine in an elderly patient. Geriatr Gerontol Int 21(6):540-540 DOI: 10.1111/ggi.14163.

Zlotnik, Y., Gadoth, A., Abu-Salameh, I., Horev, A., Novoa, R., Ifergane, G., 2022. Case report: anti-LGI1 encephalitis following COVID-19 vaccination. Front Immunol 12:813487 DOI: 10.3389/fimmu.2021.813487.
